# Slight Deuterium Enrichment in Water Acts as an Antioxidant: Is Deuterium a Cell Growth Regulator?

**DOI:** 10.1074/mcp.RA120.002231

**Published:** 2020-08-07

**Authors:** Xuepei Zhang, Jin Wang, Roman A. Zubarev

**Affiliations:** 1 Division of Physiological Chemistry I, Dept. of Medical Biochemistry and Biophysics, Karolinska Institutet, Stockholm, Sweden; 2 Department of Pharmacology, School of Basic Medical Sciences, Xi'an Jiaotong University Health Science Center, Xi'an, Shannxi, P.R. China; 3 SciLIfeLab, Stockholm, Sweden; 4 Department of Pharmacological & Technological Chemistry, I.M. Sechenov First Moscow State Medical University, Moscow, Russia

**Keywords:** Mass spectrometry, oxidative stress, quantification, chemoproteomics, thiol redox chemistry, deuterium effect, redox balance, deuterium-enriched water, antioxidant, oxidation-reduction balance, cell growth regulator

## Abstract

Small admixtures in water, *e.g.* of metal ions, often act as cell growth regulators. Here we report that enrichment of deuterium content in water, normally found at 8 mm concentration, two-three folds increases cell proliferation and lowers the oxidative stress level as well. Acting as an anti-oxidant, deuterium-enriched water prevents the toxic effect of such oxidative agents as hydrogen peroxide and auranofin. This action is opposite to that of deuterium depletion that is known to suppress cell growth and induce oxidative stress in mitochondria. We thus hypothesize that deuterium may be a natural cell growth regulator that controls mitochondrial oxidation-reduction balance. Because growth acceleration is reduced approximately by half by addition to water a minute amount (0.15%) of ^18^O isotope, at least part of the deuterium effect on cell growth can be explained by the isotopic resonance phenomenon. A slight (≈2-fold) enrichment of deuterium in water accelerates human cell growth. Quantitative MS based proteomics determined changes in protein abundances and redox states and found that deuterium-enriched water acts mainly through decreasing ROS production in mitochondria. This action is opposite to that of deuterium depletion that suppresses cell growth by inducing oxidative stress. Thus deuterium may be a natural cell growth regulator that controls mitochondrial oxidation-reduction balance. The role of isotopic resonance in this effect was validated by further experiments on bacteria.

## Abstract (precis)

A slight (≈2-fold) enrichment of deuterium in water accelerates human cell growth. Quantitative MS based proteomics determined changes in protein abundances and redox states and found that deuterium-enriched water acts mainly through decreasing ROS production in mitochondria. This action is opposite to that of deuterium depletion that suppresses cell growth by inducing oxidative stress. Thus deuterium may be a natural cell growth regulator that controls mitochondrial oxidation-reduction balance. The role of isotopic resonance in this effect was validated by further experiments on bacteria.

## Graphical Abstract

**Figure:**
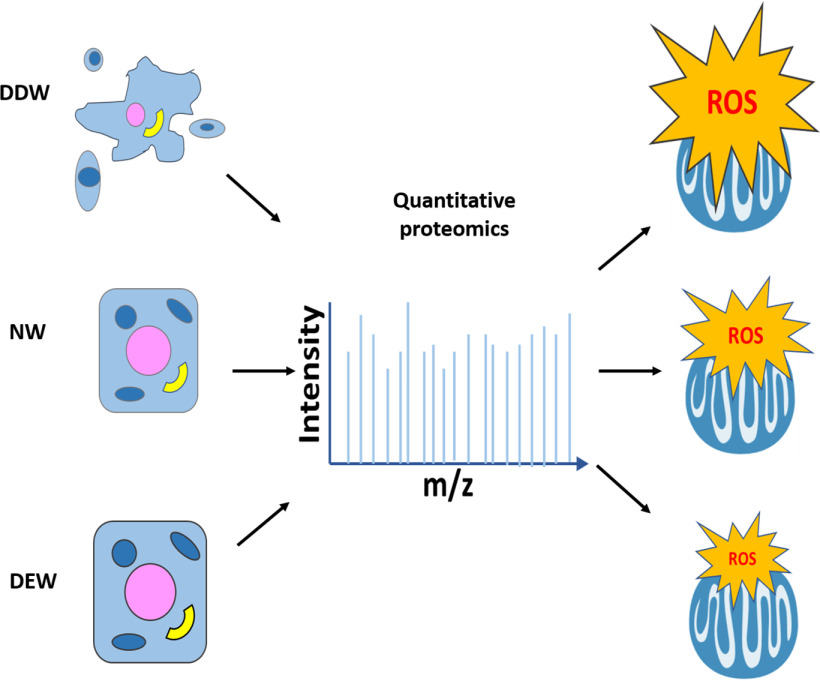

**Highlights**

- Deuterium enrichment to 350 ppm accelerates cell proliferation, which is opposite to that of deuterium depletion to 80 ppm.
- The cell proliferation increases through decreasing ROS amount in mitochondria.
- Deuterium enriched water acts as an anti-oxidant.
- Deuterium may be a cell growth regulator in the interval between 80 and 350 ppm.

Deuterium (D) is the natural heavy stable isotope of hydrogen. The relative D content in natural water is on average 0.015% (150 ppm), which is equivalent to 8 mm concentration. Deuterium fractionation occurs during vapor–liquid–ice (snow) phase transitions and during sorption and filtration in natural processes, and as a result deuterium content in water varies in terrestrial conditions within the range between 79 ppm and 195 ppm (1). By many physico-chemical manipulations, including distillation, diffusion, and chemical reactions, the concentration of deuterium in water can be made arbitrary high or low. Because the discovery of deuterium by Urey *et al.* in 1931 (2, 3), the biological effects of deuterium enrichment have been examined in a host of experimental situations (4–6). Although bacteria can endure up to 90-100% D (v/v) in deuterium enriched water (DEW), very high deuterium concentrations turned out to be incompatible with highly organized life, as they slow down cellular metabolism and cause mitotic inhibition of the prophase (4). Plant cells can develop normally in up to 75% DEW, whereas animal cells – not more than 30% DEW (7).

That high concentration of deuterium has a strong biological effect is not unexpected given its factor of two mass difference with hydrogen atom. More surprising was the finding made in 1930s that relatively small deviations from normal deuterium content, such as 4-fold deuterium enrichment, have a marked effect on various organisms (8–14). The growth rate and morphology of *Spirogyra*, flatworms, and *Euglena* in 0.06% D (600 ppm DEW) water have been found significantly affected (8–10, 12). On the other hand, in the same studies fermentation reaction slowed down by ≈15%. Lockemann and Leunig studied the effect ≤0.54% DEW upon *E. coli* and *Pseudomonas* proliferation. They observed that concentrations as low as 0.04% D enhanced growth (11). In another study, *Aspergillus* grew 10% faster in 0.05% D water (15).

Second world war interrupted these early studies, and atomic bomb project classified the deuterium-related research. But starting from 1960s, open research was resumed, and in 1970s Lobyshev *et al.* discovered that the Na, K-ATPase activity increases by up to 50% at 0.04-0.05% D, *i.e.* at a 2-3-fold enrichment (13, 14, 16). In early 1990s, Somlyai *et al.* confirmed that increasing deuterium concentration to ≈600 ppm enhances the growth rate of mammalian cells (fibroblasts) (17). However, neither of the previous studies has suggested a credible mechanism for growth acceleration by DEW.

On the other side of the enrichment scale, it has been found that deuterium depleted water (DDW) with deuterium content of 20-130 ppm suppresses cell growth, inhibiting cancer cell proliferation and tumor growth (18–20). Despite dozens of research reports, most confirming this finding, the molecular mechanism of DDW growth suppression has also been missing. Recently, we have studied the antiproliferation effect of 80 ppm DDW in A549 human lung adenocarcinoma cells with modern chemical proteomics methods and found that DDW induces mitochondrial redox imbalance which leads to oxidative stress (21).

Having discovered the mechanism of growth suppression by deuterium depleted water, we asked the question whether a similar mechanism but with an opposite sign could be responsible for growth acceleration by DEW. To test this suggestion, we designed a study the overall plan of which is shown in Fig. 1. After measuring the DEW growth in three cell lines at different deuterium concentrations, the most sensitive cell line as well as the deuterium concentration of maximum growth acceleration were to be determined (Fig. 1*A*, 1*B*). Comparison of the expression proteome data on sensitive cells treated with DEW, normal water (150 ppm, NW) as well as DDW would identify the proteins specifically up- and down-regulated by DEW (Fig. 1*C*). In parallel, the change in the oxidative states of thiols of cysteine residues in cells grown in DEW or DDW medium was to be determined with redox proteomics (Fig. 1*D*). Comparison of the results from these two complementary proteomics techniques would identify most affected proteins (Fig. 1*E*), which could uncover cellular pathways involved in DEW action (Fig. 1*F*). The hypothesis related to the DEW mechanism was then to be formulated and validated by additional tests (Fig. 1*G*). Finally, if the hypothesis was confirmed, we could consider a general model of regulation of cellular growth by deuterium concentration (Fig. 1*H*).

**Fig. 1.**
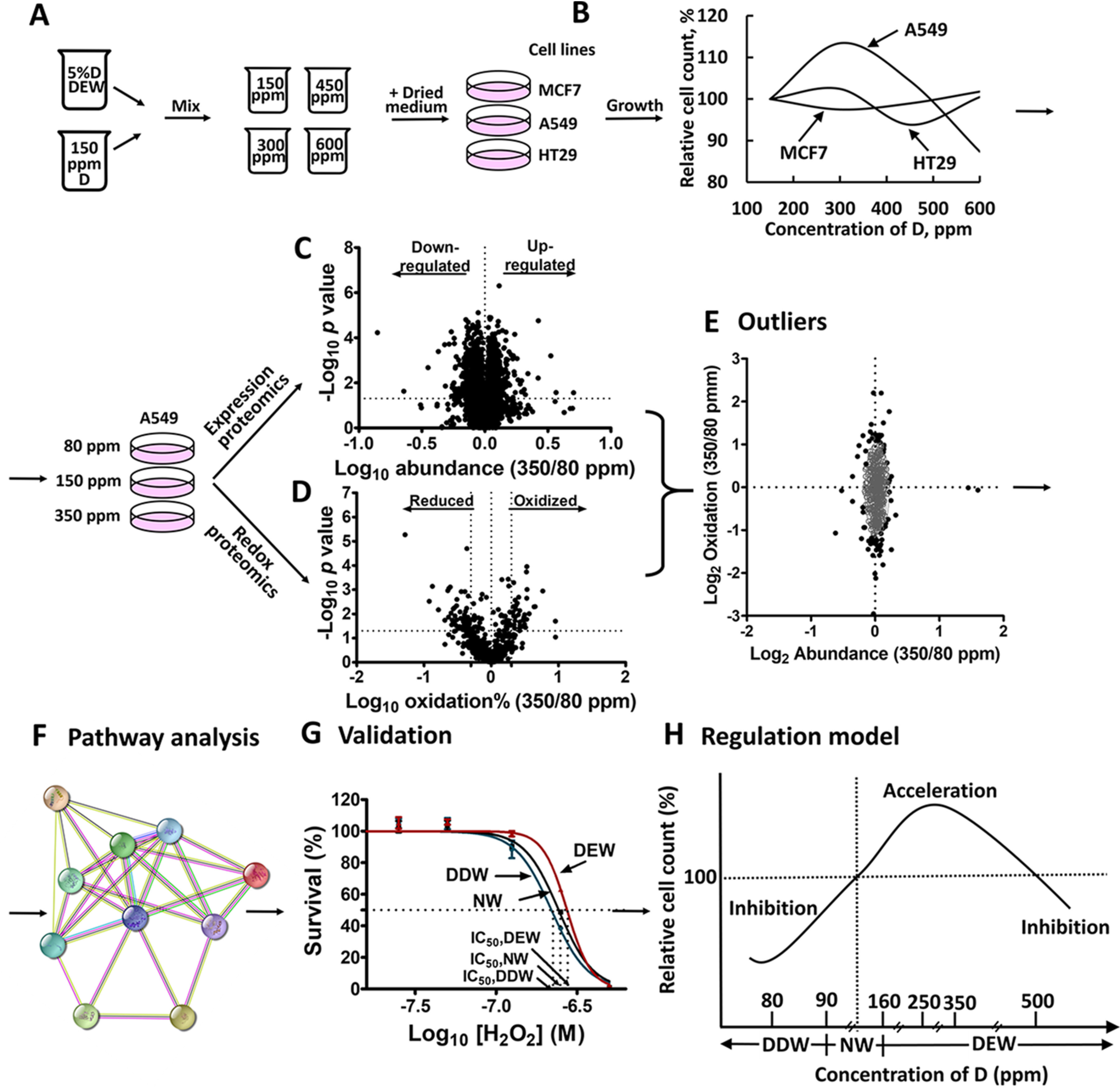
**The layout of the proteomics-based characterization of D action as a cell growth regulator.** *A*, DDW with varying deuterium concentration was prepared by mixing NW (150 ppm D) and 5% D DEW in different proportions. MCF 7, A549 and HT29 cells were grown in a DEW medium. *B*, Measurement of the cell lines responses to DEW. *C*, Identification by FITExP analysis of the most regulated by DEW proteins compared with NW and DDW. *D*, Measurement by redox proteomics of oxidation-reduction imbalance caused by DEW compared with DDW. *E*, Summary of the proteomics results reveals proteins mostly likely involved in D action. Pathway analysis of DEW action mechanism (*F*) and its validation by additional experiment (*G*). *H*, Proposed a regulation model.

## EXPERIMENTAL PROCEDURES

### 

#### 

##### Experimental Design and Statistical Rationale

The expression and redox proteomics data were obtained by nanoLC-MS/MS. To make appropriate statistical analysis, all treatments were performed in 3 biological replicates. Each set of TMT10 labeling samples were combined and fractionated into 12 fractions. In total, 54 TMT labeling samples including 4 set of TMT10 and 6 sets of iodoTMT6 labeled samples were analyzed. NanoLC-MS/MS experiments were performed with a 120 min LC gradient. In each experiment, separate controls treated with NW were included. Samples were analyzed in random order to reduce the “order of injection” effect. For A549 cells, the expression proteomics data for 80 ppm DDW were from our previous study (21). The cells were grown in 80 ppm DDW/NW, or treated with the control drugs. The data for 3 replicates of DDW samples and 1 NW sample (for normalization) were selected randomly for further analysis. The expression proteomics samples for DEW were in another TMT10 set and only the 3 replicates of cells grown in 350 ppm DEW and the 4 replicates of cells grown in NW (1 for normalization and the other 3 for comparative analysis) were used for the further study. HT29 cells were grown in 100 ppm DDW or 450 ppm DEW with 3 replicates and in NW with 4 replicates (3 for comparative analysis and 1 for normalization). In redox proteomics experiments, the cells were cultured in DDW, DEW and NW in triplicates. Quality check was performed by calculating the variation (CV) between the replicates.

##### Preparation of Water with Different Deuterium Content

DDW was prepared as described previously (21). DEW with different deuterium enrichment was obtained by mixing the corresponding volumes of NW and 5% D DEW. Water with different concentration of ^18^O were prepared by mixing the corresponding volumes of 25 ppm DDW, NW, 5% D DEW and 10% ^18^O water (150 ppm D). To obtain homogeneous mixing at molecular level, the mixed water was first shaken for 48 h, and then heated to 70 °C and kept for 30 min followed by cooling to room temperature. The heating-cooling cycle was repeated for another 4 cycles.

##### Cell Growth Measurements

MCF7, A549, and HT29 cell lines (originally obtained from ATCC) were grown in Dulbecco's Modified Eagle's Medium (DMEM, Thermo Fisher Scientific, Waltham, MA 11685260) supplemented with 10% heat-inactivated fetal bovine serum (FBS, Thermo Fisher Scientific 11560636), 1% penicillin/streptomycin (15140-122, Invitrogen, Waltham, MA) and 2 mm l-glutamine (17-605E, Fisher Scientific) in a humidified atmosphere at 37 °C in 5% CO_2_. DDW, NW and DEW were used to prepare culture medium by dissolving DMEM-high glucose powder (D5648, Sigma, Virginia Beach, VA), 3.7 g/L sodium bicarbonate (S5761, Sigma), 10% FBS (v/v) and 1% penicillin/streptomycin (v/v). For cell survival measurements, cells were washed with each medium twice before seeding. To avoid cell overgrowth, different numbers of cells were seeded in a 96-well plate (Sarstedt, Germany): 5,000 for A549 and 10,000 for MCF7 and HT29 cells. After 48 h growth, 10 µL of 5 g/L MTT (3-(4,5-dimethylthiazol-2-yl)-2,5-diphenyltetrazolium bromide, Thermo Fisher Scientific M6494) in PBS buffer (17-516F, Lonza) were added in each well, and the plate was incubated at 37 °C for 4 h. Then the cells were incubated at 37 °C for 14 h with 100 µL of 0.1 g/ml SDS (H5113, Promega, Madison, WI) in 0.1M HCl to dissolve formazan formed in cells after the reaction with MTT. The absorbance of formazan in each well was measured at 570 nm using Epoch microplate spectrophotometer (Agilent, Santa Clara, CA). The cell survival curves were analyzed using Prism version 5.02 for Windows (GraphPad Software, San Diego, CA), and the cell survival percentage was calculated.

##### Expression Proteomics Sample Preparation

Fifteen thousand A549 or 30,000 HT29 cells were seeded in each well of a 6-well plate (Sarstedt, Germany) and the cells were grown in DDW (80 ppm for A549 cells and 100 ppm for HT29 cells), NW or DEW (350 ppm for A549 cells and 450 ppm for HT29 cells) medium in three biological replicates. After 48 h growth, the cells were collected and lysed in 50 mm Tris (741883, Sigma) buffer and 8M urea (U5378, Sigma) at pH 8.5, 1% SDS, and protease inhibitor (5892791001, Sigma). After protein reduction using 8 mm 1,4-Dithiothreitol (DTT, Sigma 10708984001) and alkylation using 25 mm iodoacetamide (IAA, Sigma I1149), the proteins were precipitated using cold acetone at −20 °C overnight. Then the proteins were dissolved in 50 mm HEPES buffer at pH 8.0 containing 8 M urea and digested with Lysyl Endopeptidase (Lys C, Wako Chemicals GmbH 125-05061, Neuss, Germany) (1:75, enzyme to protein ratio) at 30 °C for 6 h and trypsin (V5111, Promega) (1:50, enzyme to protein ratio) at 37 °C overnight. After labeling using TMT-10 reagent (90110, Thermo Fisher Scientific), desalting using C18 Sep-pak (WAT054960, Waters, Milford, MA) and multiplexing, the peptides samples were fractionated using a Dionex Ultimate 3000 UPLC system (Thermo Fisher Scientific) with a Xbridge Peptide BEH C18 column (Thermo Fisher Scientific) (length, 25 cm; inner diameter, 2.1 mm; particle size, 3.5 μm; pore size, 300 Å; Waters), at a flow rate of 200 µL/min. Fractionation was performed using a binary solvent system consisting of 20 mm NH_4_OH in H_2_O (solvent A) and 20 mm NH_4_OH in acetonitrile (solvent B). Peptides were eluted with a gradient from 2% to 23% B in 42 min, to 52% B in 4 min, to 63% B in 2 min and then at 63% B for 5 min. The elution was monitored by UV absorbance at 214 nm; 96 fractions of each TMT-multiplexed sample were collected in total. Every 8 fractions were combined to a single mix and the 12 such mixes for each TMT-multiplexed sample were analyzed by nanoLC-MS/MS as customary in shotgun proteomics.

##### Redox Proteomics Sample Preparation

Fifteen thousand A549 or 30,000 HT29 cells were seeded in each well of a 6-well plate. The cells were grown in DDW (80 ppm for A549 cells and 100 ppm for HT29 cells), NW or DEW (350 ppm for A549 cells and 450 ppm for HT29 cells) medium in three biological replicates. After 48 h of growth, the cells were collected and lysed at pH 8.0 in a buffer containing 8 M urea, 50 mm HEPES, 1 mm ethylenediaminetetraacetic acid (EDTA, Sigma E9884), 1% SDS and protease inhibitors. The samples were then incubated with 4.4 mm iodoTMT-126, iodoTMT-127 and iodoTMT-128 labels (90102, Thermo Fischer Scientific) at 37 °C for 2 h, blocking free -SH and -SSH groups. After precipitation with cold methanol/chloroform, the protein pellets were dissolved in 50 mm HEPES buffer (pH = 8.0) containing 8 M urea. 10 mm Tris-(2-Carboxyethyl)phosphine hydrochloride (TCEP, Thermo Fischer Scientific T2556) was used to reduce disulfides at 50 °C for 1 h. After precipitation followed by resuspension, the samples were labeled with iodoTMT-129, iodoTMT-130 and iodoTMT-131 at 37 °C for 2 h. The labeled samples were precipitated again with cold methanol/chloroform and then re-suspended and digested with Lys C (1:75 enzyme to protein ratio) at 30 °C for 6 h and trypsin (1:50 enzyme to protein ratio) at 37 °C overnight. After desalting, the tryptic peptides were analyzed by nanoLC-MS/MS.

##### NanoLC-MS/MS Analysis

NanoLC-MS/MS analyses were performed on an Orbitrap Elite mass spectrometer (Thermo Fisher Scientific). The instrument was equipped with an EASY ElectroSpray source and connected online to an Ultimate 3000 nanoflow UPLC system. The samples were pre-concentrated and further desalted online using a PepMap C18 nano-trap column (length - 2 cm; inner diameter - 75 μm; particle size - 3 μm; pore size - 100 Å; ThermoFisher Scientific) with a flow rate of 3 µL/min for 5 min. Peptide separation was performed on an EASY-Spray C18 reversed-phase nano-LC column (Acclaim PepMap RSLC; length - 50 cm; inner diameter - 2 μm; particle size - 2 μm; pore size – 100 Å; Thermo Scientific) at 55 °C and a flow rate of 300 nL/min. Peptides were separated using a binary solvent system consisting of 0.1% (v/v) FA, 2% (v/v) ACN (solvent A) and 98% ACN (v/v), 0.1% (v/v) FA (solvent B). They were eluted with a gradient of 4–26% B in 120 min, and 26–95% B in 10 min. Subsequently, the analytical column was washed with 95% B for 5 min before re-equilibration with 4% B. The mass spectrometer was operated in the positive ion mode with data-dependent acquisition of MS/MS spectra and a dynamic exclusion time of previously selected precursor ions of 30 s. Mass spectra were acquired in a mass-to-charge (*m*/*z*) range of 375–1500 with a resolution of 120,000 at *m*/*z* 200. Automatic gain control target was set to 3 × 10^6^ with a maximum injection time of 100 ms. In every survey mass spectrum, up to 17 most abundant peptide peaks, with exclusion of singly-charged ions, were selected for higher-energy collision dissociation with normalized collision energy value set at 33. The ion selection abundance threshold was set at 0.1%. The MS/MS spectra were acquired at a resolution of 60,000, with a target value of 2 × 10^5^ ions or a maximum injection time of 120 ms. The fixed first *m*/*z* was 100, and the isolation window was 1.2 *m*/*z* units.

##### Proteomics Data Analysis

The MS/MS data were searched by the Andromeda search engine in MaxQuant 1.5.6.5 software using the “Specific Trypsin/P, Lyc/P” digestion mode with maximum two missed cleavages. Peptide identification was based on a search with mass tolerance of the precursor ion of 20 ppm (initial search), 10 ppm (second search) and fragment ion mass tolerance of 0.5 Da. A minimum peptide length of 6 amino acids was allowed, and the false discovery rate was 0.01 for proteins and peptides. The MS/MS spectra were searched against the Uniprot human database (UP000005640_9606 and UP000005640_9606_additional, containing 90,482 entries, last modified on January 26, 2019) combined with 262 common contaminants and concatenated with the reversed sequences. All known contaminants were removed from the search results. “Match between the runs” option was used to improve the identification efficiency. Carbamidomethyl (C) (only expression proteomics) was set as a fixed modification, whereas variable modifications were N-terminal acetylation, oxidation (M) as well as deamidation (NQ). Quantification was based on TMT10 reporter ions in MS/MS for expression proteomics and iodoTMT for redox proteomics. Only proteins quantified with at least two peptides were considered for quantitation.

##### Principal Component Analysis

Principal component analysis (PCA) and orthogonal projections to latent structures discriminant analysis (OPLS-DA) were performed using SIMCA 15.0 (Umetrics, Malmö, Sweden). For expression proteomics samples, TMT10 reporter abundance values were normalized to the TMT channel representing growth in NW. For redox proteomics data, the iodoTMT6 MS/MS reporters' intensities were used to calculate the oxidation percentages and the oxidation percentage of each protein in different conditions was used for PCA.

Model performance was assessed via the cumulative correlation coefficient (*R* ^2^ × [cum]), with predictive performance being based on 7-fold cross-validation (*Q* ^2^[cum]). The *p* value were calculated from the variance of cross-validated residuals (CV-ANOVA).

##### Network Analysis

Online STRING v10.5 tool (http://string-db.org) was used to map DEW-specific, significantly regulated proteins onto protein-protein interaction networks. Medium confidence threshold (0.4) was used to define protein-protein interactions. The in-built gene enrichment analysis with the whole genome as a background was used to identify enriched gene ontology terms and KEGG pathways.

##### Biochemical Analysis of ROS Levels

A549 cells were seeded in a 96-well plate and treated with 3 μm auranofin for 24 h. Then the medium was changed to DEW medium or NW medium with different concentrations of N-acetyl-cysteine (NAC). After another 24 h, the cells were counted using the MTT assay. For studying the combined effect of DDW/DEW and hydrogen peroxide (H_2_O_2_)/auranofin, 5000 A549 cells were seeded in each well of a 96-well plate, and after 48 h growth in the corresponding medium, cells were treated with different concentrations of H_2_O_2_ or auranofin for 24 h, followed by cell counting. For cellular ROS level measurement, 5000 A549 and 10,000 HT29 cells were seeded in each well of a 96-well plate and grown in DDW, NW, DEW medium or/and treated with 3 μm auranofin. After 24 h, the cells were washed with PBS twice and 100 µL 20 μm DCF-DA in PBS was added into each well. After 30 min incubation at 37 °C in darkness, the fluorescence intensity in each well was measured by Infinite^®^ M200 PRO (Tecan, Männedorf, Switzerland). The excitation and emission wavelengths were 485 nm and 535 nm, respectively. In parallel, cells were counted using the MTT assay described as above. The fluorescence intensity in each well was normalized to the average fluorescence intensity of live cells.

##### Measurement of Cellular Nascent Proteins in A549 and HT29 Cells Treated with DEW

Five thousand A549 and 10,000 HT29 cells were seeded in each well of a 96-well plate and grown in DDW, NW or DEW medium in 10 biological replicates. After 48 h, the levels of cellular nascent proteins were measured using Click-iT^®^ Plus OPP Protein Synthesis Assay Kits (C10456, Thermo Fisher Scientific) according to manufacturer's instructions.

##### E. coli BL21 Growth

*E. coli* BL21 strain (originally from Thermo Fisher Scientific) was grown on plates with LB agar (Sigma). A single colony was transferred to a tube containing 6 ml of normal M9 media and grown for 36 h. These bacteria were further diluted (1:500) into tubes containing medium with different deuterium concentrations and put into honeycomb well plates (BioScreen, Helsinki, Finland) in 20 biological replicates. Bacterial density at 37 °C was monitored by automatically measuring light diffraction during agitation-assisted growth in the BioScreen C incubator for 48 h.

## RESULTS

### 

#### 

##### 300–450 Ppm DEW Promotes Growth of Cancer Cells

**Fig. 2.**
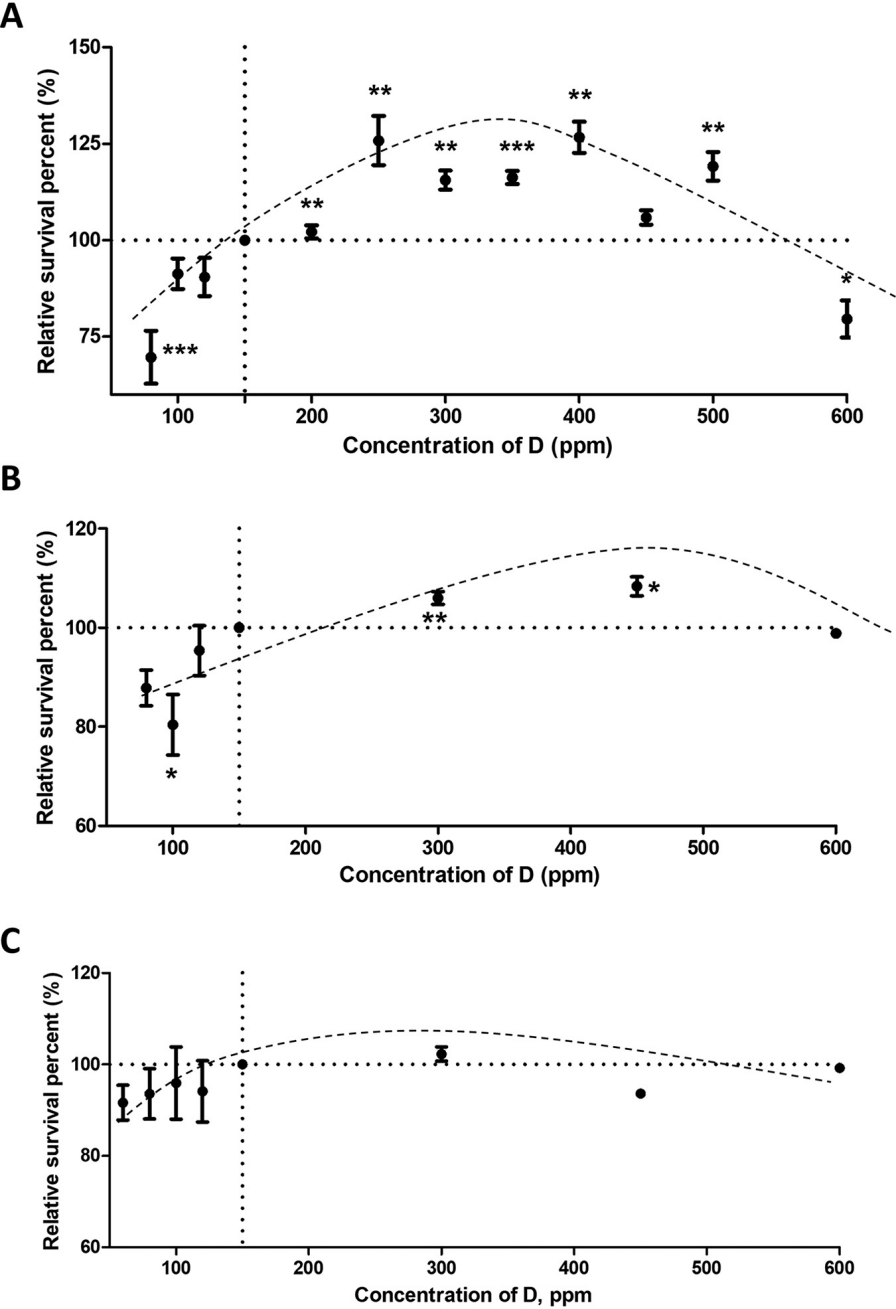
Measurement of A549 (*A*) HT29 (*B*) and MCF7 (*C*) cells response to DEW and determination of the deuterium concentration giving maximum effect. The figure shows mean ± S.E. of three independent experiments, single measurement, *p < 0.05, **p < 0.005, ***p < 0.0001 in two-tailed unparied *t* test.

Three types of human cells: breast adenocarcinoma MCF7, adenocarcinomic alveolar basal epithelial A549 cells, and colorectal adenocarcinoma HT29 cells were selected for this study. The cells grew for 48 h in DEW with deuterium concentration of 300 ppm, 450 ppm, 600 ppm and in NW as a control. The HT27 and MCF7 cells responded weakly to DEW, showing a <15% increase in proliferation rate. The A549 cells turned out to be the most responsive to DEW (Fig. 2, supplemental Fig. S1), with 300 ppm D DEW enhancing cell proliferation by 25% (*p* < 0.01). However, like drug sensitivity and resistance in cancer research (22), understanding the reasons for cell type dependence of the DEW effect is a challenge. Afterward, we narrowed the DEW concentration step to 50 ppm and tested the range 150–500 ppm. The cells proliferation was enhanced by 10–25% compared with NW in the interval of between 250 ppm and 500 ppm D. As a middle point of this interval, 350 ppm DEW was selected for further studies. At 600 ppm, a 20–25% decrease in cell proliferation was observed, consistent with deuterium toxicity at higher concentrations. As a negative control for suppressed growth, 80 ppm DDW was applied.

##### Effect of DDW on Protein Abundances

**Fig. 3.**
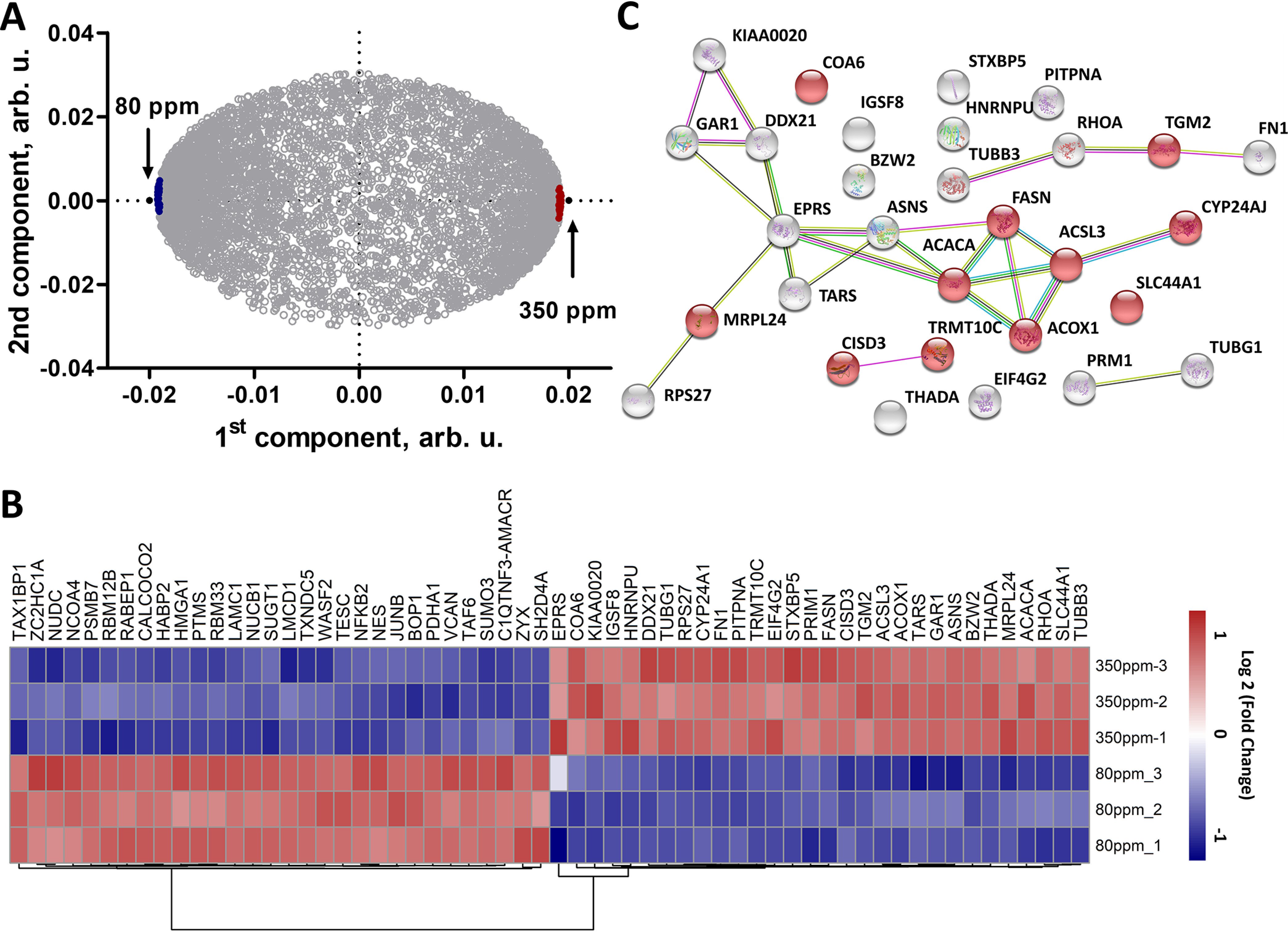
**Expression proteomics analysis at the deuterium concentration of maximum acceleration.** *A*, OPLS-DA of protein abundances for different treatments. *B*, Heat map of top 30 specifically up and down-regulated proteins in DEW treatment. *C*, Interaction network of top 30 up-regulated proteins in cells treated with DEW. Proteins marked with red color are located at mitochondria.

In total, 6082 proteins common for all three replicates of all samples were identified and quantified in A549 cells treated with DDW and DEW (supplemental Table S1). OPLS-DA was applied to analyze the difference between the two conditions (Fig. 3*A*). The top 30 up- and down-regulated proteins (top 0.5% of the common proteins) in cells grown in 350 ppm DEW were selected according to their “VIP predictive values” (Fig. 3*B*, supplemental Table S2) and classified using STRING (Fig. 3*C*, supplemental Table S3). Of the most up-regulated proteins, 11 are in mitochondria, and the fatty acid metabolism and biosynthesis were found as the most enriched biological processes. Fatty acids, stored in cells as triglycerides, are an important source of energy, because they are both reduced and anhydrous (23). Moreover, fatty acids are substrates of complex I and III via NADH and FADH2–ETF/ETF-QOR, respectively. Because fatty acid oxidation is the source of mitochondrial ROS production (24), this result hinted that DEW may act through imbalance of cellular redox equilibrium, similarly to DDW (21), but in the opposite direction. It should be noted that the most regulated proteins are different from those in our previous study (21) because of the different control (DDW in the current study and NW as well as control drugs in the previous study).

##### DEW Affects Cellular Redox Balance

Oxidative stress is defined as an imbalance between the production of free radicals and reactive metabolites, such as oxidants or reactive oxygen species (ROS), and their neutralization (detoxification) on the other hand (25). During the last two decades, extensive research has revealed that oxidative stress can lead to chronic inflammation, which in turn could mediate other chronic diseases, including cancer, diabetes, cardiovascular, neurological, and pulmonary diseases (26). On the other hand, ROS are not toxic *per se*; being generated through a variety of extracellular and intracellular processes, they act as signal mediators in growth, differentiation, progression, and cell death (27).

**Fig. 4.**
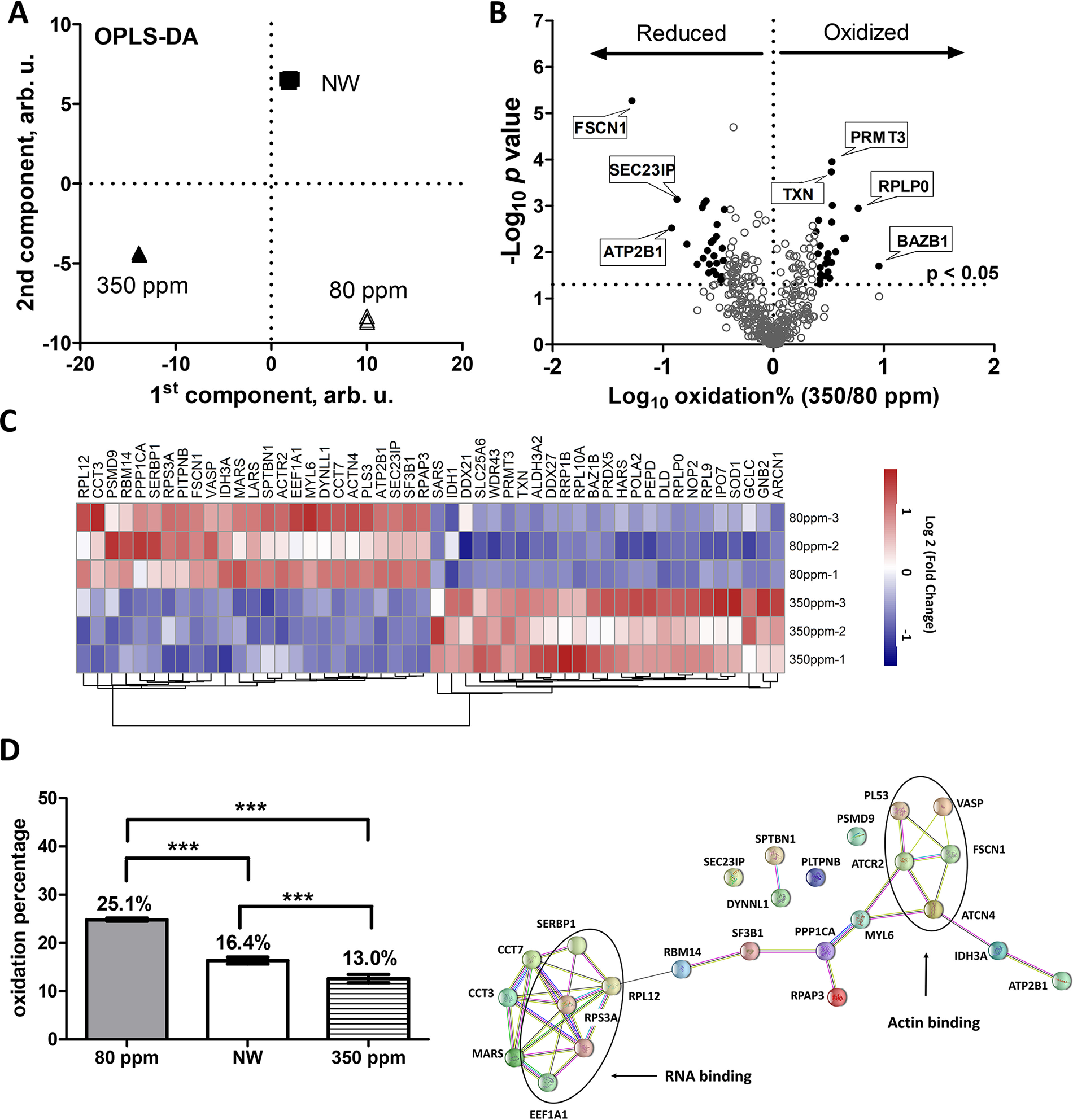
**Redox proteomics analysis of the DEW effect.** *A*, OPLS-DA of redox datasets. *B*, Volcano plot for cells treated with DEW compared with DDW. Line indicate a p value of 0.05 (-log_10_ = 1.3) in two-tailed unparied *t* test. *C*, 25 most oxidized and reduced proteins according to OPLS-DA *D*, Average oxidation levels of cells treated with NW, DEW and DDW. *E*, Interaction network of top 25 reduced proteins in cells treated with DEW. *D* shows mean ± S.E. of nine independent experiments with triplicate measurements, ***p < 0.005 in two-tailed unparied *t* test.

To understand better how DEW affects redox balance in sensitive cells, redox proteomics analysis was performed. Such analysis typically involves quantifying the fraction of free cysteine thiols and S-sulfhydration (-SSH) as opposed to the oxidized thiols in disulfide bonds, S-nitrosylation (SNO), etc. (28–30). In our analysis, 3196 proteins with cysteines were identified in total (supplemental Table S4). Of these, 426 proteins with oxidation ratios quantified in all three replicates of all samples were considered for further analysis (supplemental Table S5). OPLS-DA model showed good clustering of the data by treatments (Fig. 4*A*). The first component correlated with the deuterium content in the media, whereas the second one separated NW from the media with deviating deuterium content. In general, we observed that DEW significantly decreased the overall oxidation level of proteins compared with both DDW and NW (Fig. 4*B*). The top 25 significantly (0.05 < p) oxidized and reduced proteins (Fig. 4*C*, supplemental Table S6) were analyzed using STRING. Notably, 10 of the most oxidized proteins are located at mitochondria (supplemental Table S7), in line with expression proteomics data. Moreover, many of the 25 most reduced proteins in cells grown in DEW medium are clustered in the processes of RNA binding and actin binding (Fig. 4*E*).

##### Summary of Proteomics Results

**Fig. 5.**
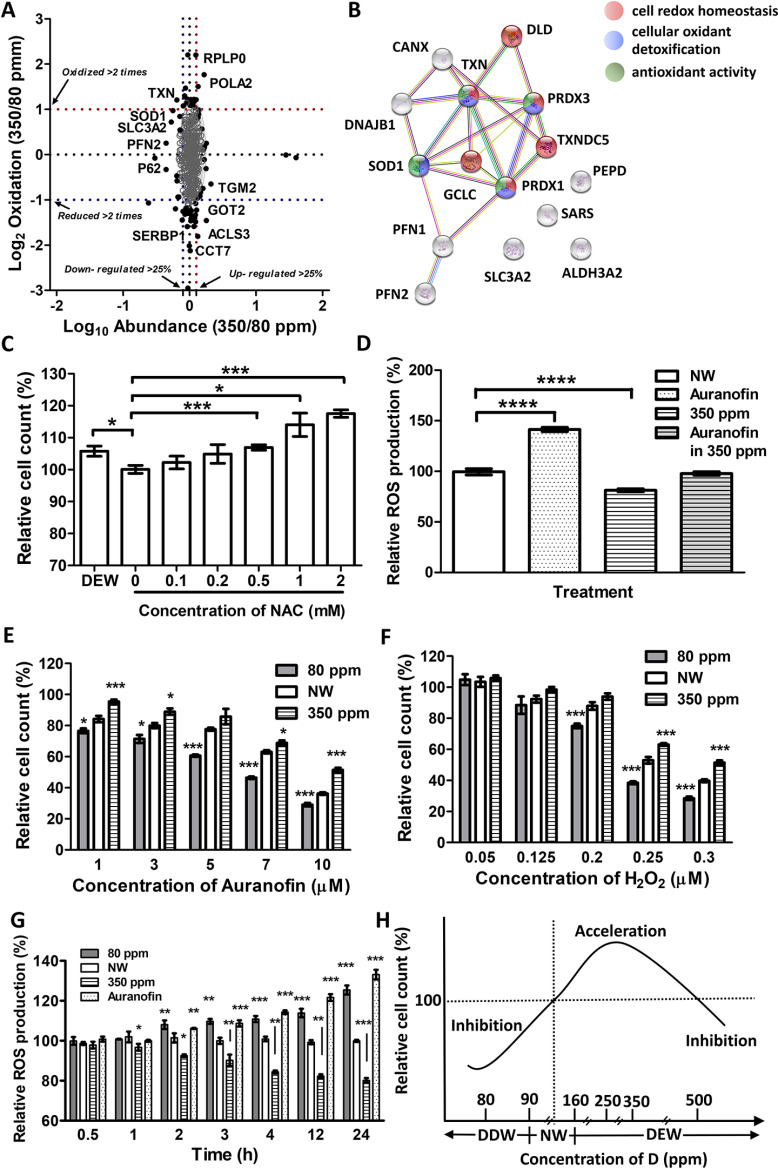
**Proposed mechanism of DEW action and its validation.** *A*, Comparison of expression and redox proteomics results. *B*, Interaction network of proteins most oxidized and down-regulated in-volved in DEW action mechanism ac-cording to outliers of proteomics analyses. *C, A*nti-oxidative effects comparison between DEW (350 ppm) and different concentrations of NAC. *D*, Relative production of ROS in cells grown in DEW (350 ppm) or/and treated with 3 μm auranofin. Responses of cells grown in DDW or DEW to auranofin (*E*) or H_2_O_2_ (*F*) treatment. *G*, Dynamic ROS production in cells grown in DEW, DDW or treated with 3 μm auranofin. *H*, Model of D regulation for cell growth. *C-G* show mean ± S.E. with four replicates measurements. *p < 0.05, **p < 0.01, ***p < 0.005 in two-tailed unparied *t* test.

The summary of expression and redox proteomics is given in Table I. The proteins identified in both strategies were selected for further analysis (Fig. 5*A*). The proteins which exhibited ≥25% abundance changes or ≥2 times modulation of the oxidation level were selected. In STRING analysis, they highlighted ribosomal-associated activities (supplemental Table S8), regulation of cellular amide metabolic process (supplemental Table S9), actin process and function (supplemental Table S10). The down-regulated and oxidized proteins were enriched in cellular reduction-oxidation activities (Fig. 5*B*, supplemental Table S11). PRDX1, PRDX3, DLD, TXNDC5, GCLC and TXN are clustered in biological process of cell redox homeostasis (Fig. 5*B*). PRDX1 and PRDX3 are members of peroxiredoxin family, which catalyze the reduction of hydrogen peroxide and organic hydroperoxides to water and alcohols, respectively. Peroxiredoxins play roles in cell protection against oxidative stress by detoxifying peroxides and as a sensor of hydrogen peroxide-mediated signaling events (31, 32). DLD is an integral component of several multienzyme systems involved in many cellular processes, such as the tricarboxylic acid (TCA) cycle (33). TXNDC5 is a member of the protein disulfide isomerase family, acting as a chaperone of endoplasmic reticulum. In addition, it has been reported that TXNDC5 is up-regulated in several cancers (34, 35). GCLC is the subunit of GCL and possesses all the catalytic activity of the enzyme, which regulates GSH synthesis (36). TXN, thioredoxin, participates in various redox reactions through the reversible oxidation of its active center dithiol to a disulfide and catalyzes dithiol-disulfide exchange reactions (37, 38). In addition, it plays a role in the reversible S-nitrosylation of cysteine residues in target proteins, and thereby contributes to the response to intracellular nitric oxide (39). Moreover, TXN, PRDX1, PRDX3 and SOD1 are related to cellular oxidant detoxification (biological process) and antioxidant activity (molecular function). SOD1, superoxide dismutase [Cu-Zn], is one of three human superoxide dismutases and is pivotal in ROS release during oxidative stress (40). The oxidation state changes in common identified peptides were also studied (supplemental Table S12) and many key peptides displayed opposite oxidation profiles in cells grown in DDW and DEW. Table II listed the oxidized cysteine sits in the common peptides of these 7 proteins highly related to cellular redox activity (supplemental Fig. S1). Many of the sites with modulated oxidative state have been reported earlier (41–47), but Cys_211_ in PRDX3, Cys_381_ in TXNDC5 and Cys_553_ in GCLC are new potentially important sites.

**Table I:** Summary of chemical proteomics results for regulated proteins

Protein Names	Gene names	Log_10_ (Oxidation, DEW/DDW)	Log_2_ (Expression, DEW/DDW)
Profilin-2	PFN2	1.29	0.7
4F2 cell-surface antigen heavy chain	SLC3A2	2.05	0.76
Superoxide dismutase [Cu-Zn]^a^	SOD1	2.65	0.78
Thioredoxin^a^	TXN	3.34	0.83
Calnexin	CANX	1	0.85
Profilin-1	PFN1	2.33	0.86
Thioredoxin domain-containing protein 5^a^	TXNDC5	1.11	0.86
Peroxiredoxin-1	PRDX1	1.58	0.87
Glutamate--cysteine ligase catalytic subunit^a^	GCLC	2.94	0.91
Xaa-Pro dipeptidase	PEPD	3.66	0.93
Thioredoxin-dependent peroxide reductase, mitochondrial*	PRDX3	2.31	0.93
Fatty aldehyde dehydrogenase^a^	ALDH3A2	4.35	0.94
Dihydrolipoyl dehydrogenase, mitochondrial^a^	DLD	3.41	0.96
Serine--tRNA ligase, cytoplasmic	SARS	3.13	0.96
DnaJ homolog subfamily B member 1	DNAJB1	9.05	0.97
Peroxiredoxin-5, mitochondrial^a^	PRDX5	2.98	1.01
60S ribosomal protein L10a	RPL10A	3.23	1.03
Probable ATP-dependent RNA helicase DDX27	DDX27	3.07	1.05
Protein arginine N-methyltransferase 3	PRMT3	3.39	1.07
39S ribosomal protein L11, mitochondrial^a^	MRPL11	2.87	1.08
Tyrosine-protein kinase BAZ1B	BAZ1B	9.02	1.1
Guanine nucleotide-binding protein G(I)/G(S)/G(T) subunit beta-2	GNB2	3.36	1.1
DNA polymerase alpha subunit B	POLA2	4.5	1.14
Nucleolar RNA helicase 2	DDX21	2.64	1.15
Phosphoserine aminotransferase	PSAT1	1.77	1.2
Eukaryotic translation initiation factor 3 subunit M	EIF3M	1.12	1.23
>60S acidic ribosomal protein P0	RPLP0	5.86	1.24
Tissue factor pathway inhibitor 2	TFPI2	1.18	1.25
Coiled-coil-helix-coiled-coil-helix domain-containing protein 2^a^	CHCHD2	1.29	1.29
Plasminogen activator inhibitor 1 RNA-binding protein	SERBP1	0.34	0.54
26S proteasome nonATPase regulatory subunit 9	PSMD9	0.3	0.81
Tropomyosin beta chain	TPM2	0.39	0.85
Vasodilator-stimulated phosphoprotein	VASP	0.24	0.89
Myosin light polypeptide 6	MYL6	0.29	0.9
Phosphatidylinositol transfer protein beta isoform	PITPNB	0.31	0.95
Spectrin beta chain, nonerythrocytic 1	SPTBN1	0.2	0.96
Fascin	FSCN1	0.05	0.97
SEC23-interacting protein	SEC23IP	0.13	0.99
Alpha-actinin-4	ACTN4	0.23	1.02
Heterogeneous nuclear ribonucleoprotein Q	SYNCRIP	0.94	1.04
T-complex protein 1 subunit gamma	CCT3	0.26	1.09
Leucine--tRNA ligase, cytoplasmic	LARS	0.23	1.09
Fragile X mental retardation syndrome-related protein 1	FXR1	0.99	1.1
Lysophospholipid acyltransferase 7	MBOAT7	0.4	1.17
Aspartate aminotransferase, mitochondrial*	GOT2	0.47	1.22
60S ribosomal protein L24	RPL24	0.9	1.25
Major prion protein	PRNP	0.73	1.28
Long-chain-fatty-acid--CoA ligase 3	ACSL3	0.23	1.29
Protein-glutamine gamma-glutamyltransferase 2	TGM2	0.52	1.38

^a^Proteins located at mitochondria.

**Table II:** Changes in oxidation states of cysteines in peptides of identified proteins and their role in cellular redox processes

Sequence	Protein Names	Gene Names	Oxidation %, DEW/DDW	*p* value	Feature Key	Ref.
LA^a^*Cys_128_*GVIGIAQ	Superoxide dismutase [Cu-Zn]	SOD1	1.89	0.019	Disulfide bond	(41)
NETLGGT^a^*Cys_80_*LNVG^a^*Cys_85_*IPSK	Dihydrolipoyl dehydrogenase	DLD	1.31	0.017	Disulfide bond	(42)
^a^*Cys_73_*MPTFQFFK	Thioredoxin	TXN	5.92	0.004	Disulfide bond; Modified residue (S-nitrosocysteine)	(43–45)
LVVVDFSATW^a^*Cys_32_*GP^a^*Cys_35_*K			1.75	0.002	Active site (Nucleophile); Disulfide bond	(45, 46)
AFQYVETHGEV^b^*Cys_211_*PANWTPDSPTIK	Thioredoxin-dependent peroxide reductase	PRDX3	2.31	0.006		
IAEVD^b^*Cys_381_*TAER	Thioredoxin domain-containing protein 5	TXNDC5	1.34	0.023		
HGEV^a^*Cys_173_*PAGWK	Peroxiredoxin-1	PRDX1	1.99	0.026	Disulfide bond	(47)
^b^*Cys_553_*SILNYLK	Glutamate--cysteine ligase catalytic subunit	GCLC	1.69	0.026		

^a^Function of the site has been reported in provided reference.

^b^New redox activity site identified in this study.

Based on the above results, we hypothesize that DEW affects the redox balance in mitochondria, reducing the overall ROS level. Thus, DEW should act as an antioxidant.

##### Validation

To test the above hypothesis, we attempted to compare the DEW antioxidative effect with that of N-acetyl cysteine (NAC), the known antioxidant. NAC counteracted (Fig. 5*C*) the suppression of cell proliferation induced by auranofin (a known oxidizing agent (48)). A similar and significant (*p* < 0.05) effect was also observed in 350 ppm DEW media, with the action like that of 0.2 mm NAC (Fig. 5*C*).

To confirm DEW effect on ROS, ROS levels in cells were assessed by a traditional biochemical assay. 3 μm auranofin treatment increased ROS production by ≈40% (*p* < 0.0001) compared with NW, in 350 ppm DEW ROS levels decreased by ≈15% (*p* < 0.0001, Fig. 5*D*). In cells growing in 350 ppm DEW and treated with 3 μm auranofin, the ROS level remained largely the same (*p* > 0.05) as in NW without any treatment.

For better contrast, the antioxidant effect of DEW was compared with the oxidative-stress inducing action of 80 ppm DDW (21). In both cases, the cells were treated with auranofin. As the auranofin concentration increased, the difference between DEW and NW and well as DDW became increasingly more apparent. At the maximum tested auranofin concentration of 10 μm, almost twice as many cells survived in DEW than in DDW (Fig. 5*E*).

The cell survival rates after treatment with H_2_O_2_ were also much higher in DEW than DDW. In NW, the suppression effect like 10 μm auranofin was obtained with 0.3 μm H_2_O_2_ (Fig. 5*F*, supplemental Table S2). And again, twice as many cells survived in DEW than in DDW.

These results demonstrate that DEW inhibits the oxidative stress induced by oxidants whereas DDW, amplifies the oxidative stress, as we have earlier demonstrated (21). The different effect of DDW/DEW on ROS production is notable already after one hour of incubation in the corresponding media (Fig. 5*G*).

##### DEW Affects Protein Expression and Redox Balance in HT29 Cells

**Fig. 6.**
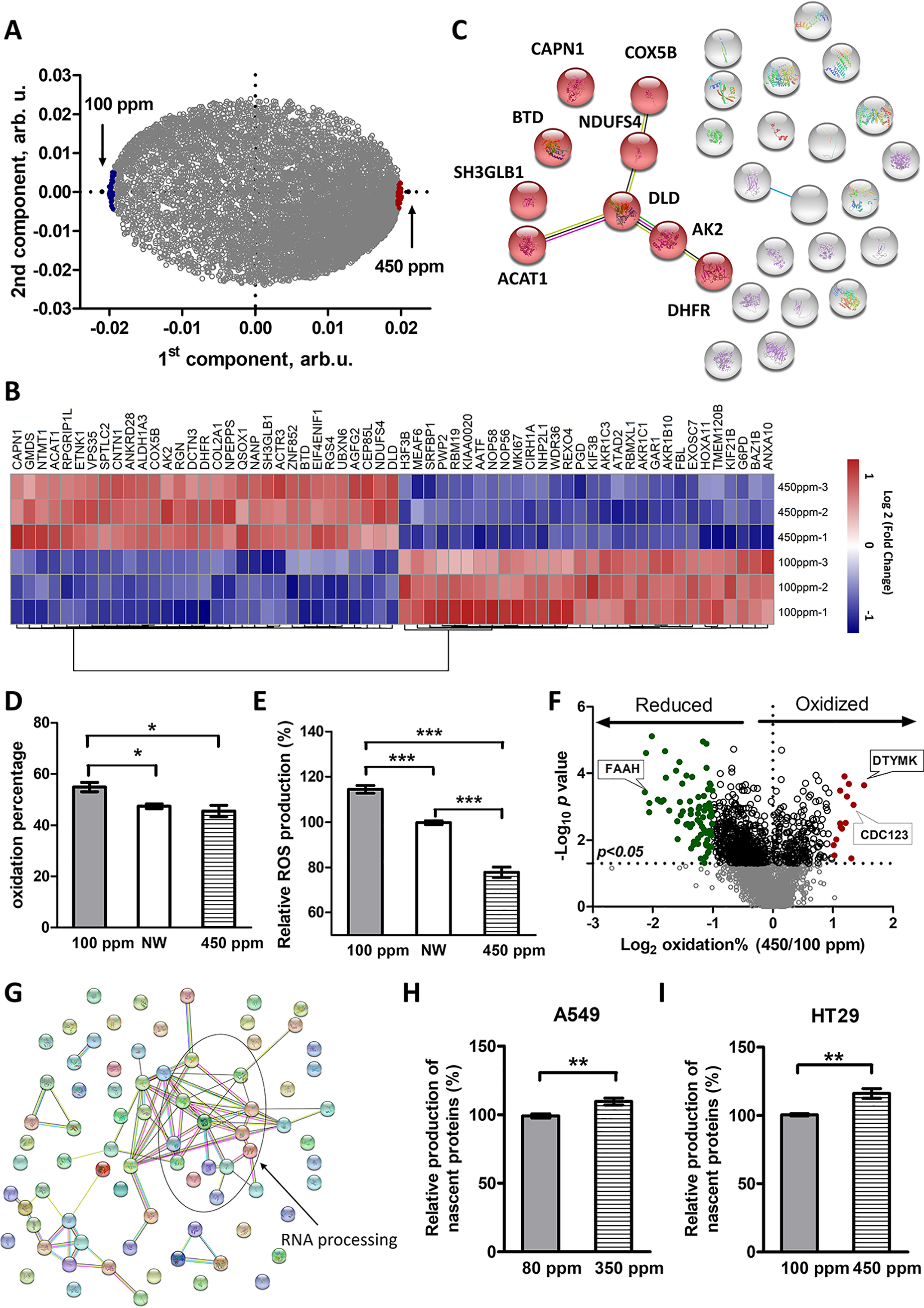
**The effect of DEW on HT29 cells.** *A*, Loading plot of an OPLS-DA model for different treatments based on protein abundances, 450 ppm DEW *versus* 100 ppm DDW. *B*, Heat map of top 30 specifically up- and down-regulated proteins. *C*, Interaction network of these proteins; molecules marked red are located at mitochondria. *D*, Average oxidation levels of proteins – average of three independent experiments, each performed in triplicates. *E*, Relative production of ROS, average of 10 independent experiments. *F*, Volcano plot for oxidation levels in proteins from DEW compared with DDW. *G*, Interaction network of significantly reduced proteins (fold change < 0.5). *H*, Relative production of nascent proteins in A549 cells, average of six independent experiments. *I*, Same in HT29 cells. Error bars correspond to standard error. *p < 0.05, **p < 0.01, ***p < 0.005 in two-tailed unpaired *t* test.

Expression and redox proteomics analyses were performed on HT29 cells to validate the above findings. Compared with A549 cells, the suppression effect of DDW and acceleration effect of DEW were less dramatic. HT29 cells growth was most inhibited (-20%) when the concentration of D was 100 ppm, and most accelerated (+10%) in 450 ppm DEW (Fig. 2*B*). Thus these deuterium concentrations were chosen for HT29 as DDW and DEW conditions, respectively. In total, 8143 proteins were identified and quantified in all 3 replicates in all samples (supplemental Table S13). OPLS-DA was applied to analyze the expression difference (Fig. 6*A*). The top 30 up- and down-regulated proteins in HT 29 cells grown in 450 ppm DEW were selected according to their “VIP predictive values” (Fig. 6*B*, supplemental Table S14) and classified using STRING (Fig. 6*C*, supplemental Fig. S3). Similar with A549 cells, 9 of the most up-regulated proteins in HT29 cells grown in 450 ppm DEW medium were located at mitochondria and oxidoreductase activity was one of the most enriched molecular functions. Redox proteomics analysis was also performed in HT29 cells (supplemental Table S15). The average oxidation level in HT29 cells grown in 450 ppm DEW was lower than in NW (but without statistical significance) and significantly lower than in 100 ppm DDW (Fig. 6*D*). This result was further confirmed by the DCF-DA assay, which showed significantly lower ROS levels in HT29 cells grown in DEW compared with NW and much lower compared with DDW (Fig. 6*E*). The proteins with most significant regulation in redox status (fold change < 0.5 or > 2, *p* < 0.05) were selected and classified. Many of the reduced proteins were enriched in RNA processing (Fig. 6*F*, supplemental Table S16), which was like A549.

In general, the HT29 results were similar to A549 results. That led us to hypothesize that DEW may accelerates cell growth by enhancing translational processes. This hypothesis was validated by finding significantly higher levels of cellular nascent proteins in A549 and HT29 cells grown in DEW compared with DDW (Figs. 6*H* and 6*I*, respectively).

##### Model of D Regulation of Cell Growth

Growth and survival of cells is strongly affected by cellular variables, such as temperature, nutrient and oxygen availability, pH and osmolarity. Because in cellular environments, these parameters are highly dynamic, microorganisms must cope with fluctuating, often nonoptimal growth conditions. As an example, calcium ions regulate over 300 biochemical reactions in the body through their role as enzyme co-factors (49). Proliferation in cell cultures of mouse 3T3 (50) or human WI-38 (51) fibroblasts was found to be inhibited by calcium concentrations below 0.5 mm, leading to arrest of cells in the late Gl phase. On the other hand, proliferation of these cell types reaches maximum at calcium levels of 0.5-1.25 mm, decreasing at higher concentrations. Therefore, calcium ion concentration is an important regulator of cellular growth (52, 53). As with every regulator, calcium is characterized by a cell-specific concentration range. Within that range, regulation takes place, with higher concentrations leading to faster growth until maximum-growth concentration is reached. Similarly, our finding is that deuterium regulates cell growth within the concentration range between 80 ppm and 250–450 ppm (Fig. 5*H*). Within that range, lower D concentration results into slower cell proliferation, whereas higher D concentration leads to faster growth.

As a cell growth regulator, deuterium acts by shifting the equilibrium in cellular mitochondrial redox processes. The exact molecular mechanism is still waiting to be explored, although for DDW action we have proposed an heuristic model (21). But on a more fundamental level, the regulation of cell growth by deuterium may be a manifestation of the more general mechanism, such as the isotopic resonance (54). The isotopic resonance phenomenon relates to the reduction of system's overall quantum-mechanical complexity at “resonance” values of isotopic abundances, which leads to faster kinetics of biochemical reactions. The basis for the isotopic resonance phenomenon is the postulate that less complex systems tend to react faster, as well as that the symmetry (defined as any condition that leads to a reduction in the number of degrees of freedom) makes systems less complex. In most isotopic resonance phenomena, symmetry is not geometric, but is achieved when “numbers match,” *e.g.* when average isotopic mass becomes commensurable with the monoisotopic mass. The isotopic resonance for normal isotopic compositions of the element C, N, and O and variable composition of D is found in the range between 250 and 350 ppm D (55), in agreement with the maximum growth of A549 cells observed in our experiments. Isotopic resonance often enhances the reaction rates, but some reactions are retarded. For instance, recently our group reported that 350 ppm D water reduces by 30% the rate of luceferin oxidation by luciferase (55). The isotopic resonance paradigm predicts that, when the system moves away from isotopic resonance, the effect (enhancement or retardation of the biochemical reaction or organism growth) should become progressively weaker. In principle, this prediction agrees well with growth suppression by DDW.

**Fig. 7.**
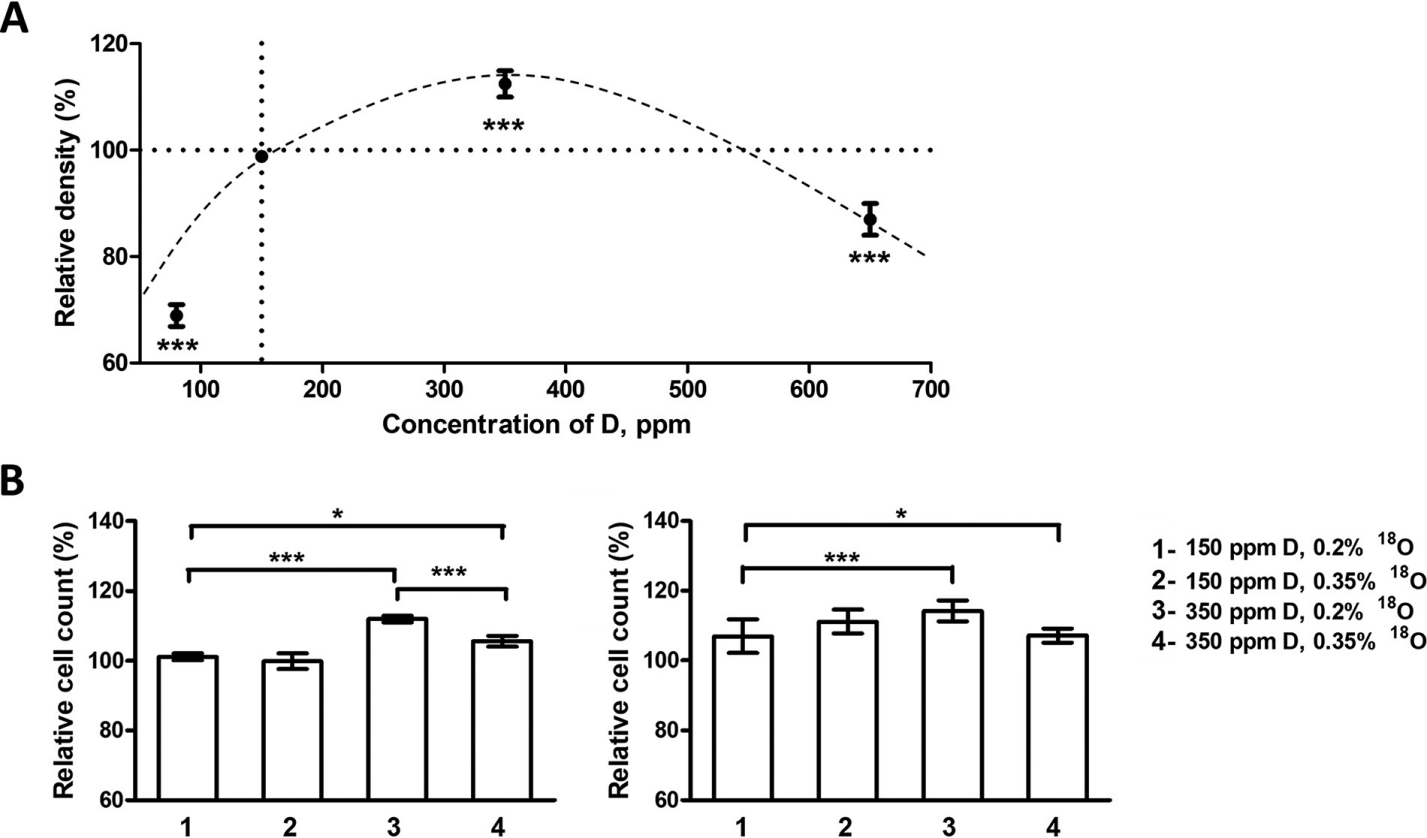
**Validation of Model of D regulation of cell growth.** *A*, BL21 *E.coli* relative cell density in response to varying deuterium content in water, average of 20 replicates. *B*, Two independent experiments, both performed in *n* = 5 biological replicates, demonstrating positive DEW effect on cell growth in presence of the isotopic resonance conditions (3 *versus* 1). Even a small change in ^18^O content destroys these conditions, greatly reducing the DEW effect (4 *versus* 3). When the isotopic resonance conditions are distant, the same change in ^18^O content does not affect cell growth (2 *versus* 1).

To test whether isotopic resonance phenomenon is at least partially responsible for the observed effects, growth rates of *E.coli* BL21 bacteria in the M9 medium with different concentration of D was analyzed (Fig. 7*A*). It was discovered that 80 ppm DDW suppresses bacterial growth, whereas 350 ppm DEW enhances it compared with NW. These measurements were repeated at two different ^18^O concentrations, ≈0.2% (normal concentration) and ≈0.35%. The modulation of ^18^O concentration was achieved by adding to growth media a small volume of water with 10% ^18^O or the same volume of NW. At 0.35% ^18^O, no isotopic resonance should be present at any deuterium concentration used. The results of two independent experiments are presented in Fig. 7*B*. Without the isotopic resonance, the growth increase because of DEW more than halved compared with on-resonance conditions at the same deuterium concentration. At normal deuterium concentration, at which the isotopic resonance conditions are more distant, the same modulation of ^18^O concentration produced little effect on cell growth. Therefore, at least part of the DEW growth acceleration effect is because of the isotopic resonance phenomenon.

## CONCLUSIONS

Here we confirmed the acceleration effect of low-level DEW on cell proliferation and discovered that, on the organelle level, it acts mainly through causing imbalance of mitochondrial redox states in the opposite direction than DDW. Thus, on metabolic level DEW acts as an antioxidant. On the protein level, DEW modulates the expression of proteins involved in fatty acid metabolism and biosynthesis, with many regulated proteins being in mitochondria. The proposed heuristic model shows that when the concentration of D is lower than the normal value of ≈150 ppm, the oxidative stress increases, slowing down cell proliferation. However, when D concentration becomes higher than normal, cell growth is promoted through inhibition of ROS production. The growth acceleration effect seems to peak in a broad range of deuterium concentrations between 250 and 500 ppm, with ≈350 ppm being the center of that interval. Incidentally or not, this is the value predicted for maximum growth acceleration by the isotopic resonance paradigm (55, 56), which is supported by several previous observations (13, 14). Because the growth acceleration effect at 350 ppm is nearly halved when the resonance conditions are destroyed, at least part of the effect must be because of the isotopic resonance. Interestingly, Lobysheva *et al.* have recently not only confirmed the effect of deuterium content in water on the function of mitochondria, but also concluded that this effect is because of water itself rather than alterations in biomolecular mechanics of the cells (57).

If this latter result is confirmed, the exact molecular mechanism of deuterium-induced growth acceleration becomes less relevant, as the isotopic resonance is a fundamental phenomenon. The situation can be compared with starvation – while denying cells or an organism energy-bearing nutrients inevitably causes growth suppression and ultimately death, the fundamental explanation is the energy conservation law, whereas at the molecular level, starvation activates a plethora of signaling and metabolic pathways. However, blocking or inhibiting these pathways would be futile to prevent cell demise. Similarly, the uncovered molecular pathways may be a manifestation of the isotopic resonance phenomenon, but not the fundamental cause of the growth modulation.

## DATA AVAILABILITY

Excel files containing the analyzed data are provided in supplemental material. The MS proteomics data have been deposited to the ProteomeXchange Consortium via the PRIDE partner repository with the data set identifier PXD018216.

## Supplementary Material

Supplemental Data

Supplementary table s1

Supplementary table s2

Supplementary table s4

Supplementary table s5

Supplementary table s12

Supplementary table s13

Supplementary table s14

Supplementary table s15

Supplementary table s16
