# Supplementary material for: Slight Deuterium Enrichment in Water Acts as an Antioxidant: Is Deuterium a Cell Growth Regulator?
Source: Mol Cell Proteomics. 2020 Aug 7;19(11):1790–804. doi: 10.1074/mcp.RA120.002231 (PMC7664117; doi:10.1074/mcp.RA120.002231)
Supplement: Supplemental Data [file 162279_1_supp_576171_qpjfdp.pdf]

Slight deuterium enrichment in water acts as an antioxidant:

is deuterium a cell growth regulator?

Xuepei Zhang, Jin Wang, Roman A. Zubarev*

Supplementary Figure captions:

Table S1. Common proteins identified in expression proteomics analysis in A549 cells grown in DEW, NW and DDW.

Table S2. Top 30 up- and down-regulated proteins in A549 cells grown in DEW and their abundance ratios in comparison with DDW.

Table S3. STRING analysis of top 30 up-regulated proteins in A549 cells (DEW vs DDW).

Table S4. Common proteins identified in redox proteomics analysis in A549 cells grown in DEW, NW and DDW.

Table S5. Common proteins identified in redox proteomics analysis in A549 cells grown in DDW and DEW.

Table S6. Top 25 oxidized and reduced proteins in A549 cells (DEW vs DDW).

Table S7. STRING analysis result of top 25 oxidized proteins in A549 cells (DEW vs DDW).

Table S8. STRING analysis result of up- regulated and oxidized outlying proteins in A549 cells.

Table S9. STRING analysis result of up- regulated and reduced outlying proteins in A549 cells.

Table S10. STRING analysis result of down- regulated and reduced outlying proteins in A549 cells.

Table S11. STRING analysis result of down- regulated and oxidized outlying proteins in A549 cells.

Table S12. Common peptides identified in redox proteomics analysis in A549 cells grown in DEW, NW and DDW.

Table S13. Common proteins identified in expression proteomics analysis in HT29 cells grown in DEW, NW and DDW.

Table S14. Top 30 up- and down-regulated proteins in HT29 cells grown in DEW and their abundance ratios compared to DDW.

Table S15. Common proteins identified in redox proteomics analysis in HT29 cells grown in DEW, NW and DDW.

Table S16. Significantly reduced (fold change < 0.5, n = 78) and oxidized (fold change > 2, n = 15) proteins identified in redox proteomics analysis in HT29 cells grown in DEW compared to DDW.

Table S17. Oxidation state of most significantly oxidized and reduced proteins in A549 cells grown in DEW, NW and DDW.

Figure S1. Oxidized percentage of cysteines in the peptides in A549 cells for DEW vs DDW.

Figure S2. Survival curves of A549 cells in DDW, NW or DEW treated with A) H_2O_2, B) auranofin.

Figure S3. STRING network analysis of significantly regulated proteins in HT29 cells grown in 450 ppm DEW vs 100 ppm DDW.

Figure S4. Redox proteomics analysis of deuterium effect on HT29 cells.

Table S3. STRING analysis of top 30 up-regulated proteins in A549 cells (DEW vs DDW).

	**Pathway ID**	**Pathway description**	**Observed gene count**	**False discovery rate**
**Biological Process (GO)**	GO.0035338	long-chain fatty-acyl-CoA biosynthetic process	3	0.03
	GO.0043603	cellular amide metabolic process	7	0.03
	GO.0001503	ossification	5	0.04
	GO.0001649	osteoblast differentiation	4	0.04
**Cellular Component (GO)**	GO.0005739	mitochondrion	11	0.009
	GO.0043232	intracellular non-membrane-bounded organelle	14	0.04
**KEGG Pathways**	1212	Fatty acid metabolism	4	0.0002
	61	Fatty acid biosynthesis	2	0.004
	1100	Metabolic pathways	8	0.02

Table S6. Top 25 oxidized and reduced proteins in A549 cells (DEW vs DDW).

Protein names	Gene names	Sequence coverage [%]	MQ Score	Oxidation ratio, DEW/DDW	p value
Tyrosine-protein kinase BAZ1B	BAZ1B	6.9	56.08	9.02	1.99E-02
60S acidic ribosomal protein P0	RPLP0	47	68.95	5.86	1.13E-03
DNA polymerase alpha subunit B	POLA2	9	24.98	4.50	5.00E-03
Fatty aldehyde dehydrogenase	ALDH3A2	30.9	84.86	4.35	5.11E-03
Xaa-Pro dipeptidase	PEPD	35.4	86.50	3.66	9.83E-03
Dihydrolipoyl dehydrogenase, mitochondrial	DLD	16.3	47.00	3.41	9.82E-04
Protein arginine N-methyltransferase 3	PRMT3	6.4	25.78	3.39	1.12E-04
Probable 28S rRNA (cytosine(4447)-C(5))-methyltransferase	NOP2	19.6	94.14	3.38	2.25E-03
Guanine nucleotide-binding protein G(I)/G(S)/G(T) subunit beta-2	GNB2	42.4	45.30	3.36	1.68E-02
Thioredoxin	TXN	77.1	60.03	3.34	1.85E-04
Ribosomal RNA processing protein 1 homolog B	RRP1B	12.2	49.88	3.25	3.63E-02
60S ribosomal protein L10a	RPL10A	38.7	59.92	3.23	1.66E-02
Isocitrate dehydrogenase [NADP] cytoplasmic	IDH1	32.6	90.61	3.14	1.07E-02
Serine--tRNA ligase, cytoplasmic	SARS	24.6	78.18	3.13	2.67E-02
Probable ATP-dependent RNA helicase DDX27	DDX27	14	68.52	3.07	1.35E-02
Peroxiredoxin-5, mitochondrial	PRDX5	48.1	46.01	2.98	1.78E-02
Glutamate--cysteine ligase catalytic subunit	GCLC	28.3	129.03	2.94	3.29E-02
Importin-7	IPO7	18.8	122.01	2.68	3.01E-02
60S ribosomal protein L9	RPL9	30.2	52.21	2.66	2.16E-02
Coatomer subunit delta	ARCN1	18	69.60	2.66	7.26E-03
Superoxide dismutase [Cu-Zn]	SOD1	25.2	15.98	2.65	3.86E-02
Nucleolar RNA helicase 2	DDX21	40.9	188.65	2.64	4.94E-02
WD repeat-containing protein 43	WDR43	21	64.34	2.57	2.05E-03
Histidine--tRNA ligase, cytoplasmic	HARS	35	107.16	2.53	1.09E-02
ADP/ATP translocase 3	SLC25A6	46	100.86	2.44	3.60E-03
Fascin	FSCN1	32.7	121.81	0.05	5.36E-06
Plasma membrane calcium-transporting ATPase 1	ATP2B1	10.7	88.23	0.12	3.03E-03
SEC23-interacting protein	SEC23IP	10.1	50.12	0.13	7.28E-04
T-complex protein 1 subunit eta	CCT7	44.2	163.21	0.16	6.72E-03
Spectrin beta chain, non-erythrocytic 1	SPTBN1	37.8	323.31	0.20	1.84E-02
Leucine--tRNA ligase, cytoplasmic	LARS	25.3	174.18	0.23	1.09E-03
Alpha-actinin-4	ACTN4	55.5	323.31	0.23	1.35E-02
Vasodilator-stimulated phosphoprotein	VASP	26.3	62.60	0.24	8.83E-04
Splicing factor 3B subunit 1	SF3B1	15.1	120.63	0.25	7.76E-04
40S ribosomal protein S3a	RPS3A	36.3	56.34	0.25	9.27E-03
Elongation factor 1-alpha 1	EEF1A1	42.4	193.04	0.26	2.81E-02
T-complex protein 1 subunit gamma	CCT3	46.5	185.96	0.26	1.81E-02
RNA-binding protein 14	RBM14	23.6	103.07	0.27	6.20E-03
Plastin-3	PLS3	52.1	244.48	0.28	5.63E-03
Myosin light polypeptide 6	MYL6	45.5	60.25	0.29	2.55E-02
Methionine--tRNA ligase, cytoplasmic	MARS	29.8	168.71	0.29	1.21E-02
26S proteasome non-ATPase regulatory subunit 9	PSMD9	29.6	43.78	0.30	3.06E-02
Phosphatidylinositol transfer protein beta isoform	PITPNB	36.2	51.53	0.31	4.57E-03
Dynein light chain 1, cytoplasmic	DYNLL1	50.6	37.12	0.31	1.75E-02
Isocitrate dehydrogenase [NAD] subunit alpha, mitochondrial	IDH3A	28.1	68.78	0.31	2.54E-03
60S ribosomal protein L12	RPL12	49.1	53.84	0.33	3.99E-02
Serine/threonine-protein phosphatase PP1-alpha catalytic subunit;Serine/threonine-protein phosphatase	PPP1CA	49.7	101.81	0.34	3.35E-02
Plasminogen activator inhibitor 1 RNA-binding protein	SERBP1	32.6	100.83	0.34	8.29E-03
Actin-related protein 2	ACTR2	26.4	85.34	0.35	1.52E-02
RNA polymerase II-associated protein 3	RPAP3	13.5	42.76	0.36	1.20E-03

Table S7. STRING analysis result of top 25 oxidized proteins in A549 cells (DEW vs DDW).

	**Pathway ID**	**Pathway description**	**Observed gene count**	**False discovery rate**
Biological Process (GO)	GO.0043603	cellular amide metabolic process	8	0.00317
	GO.0006518	peptide metabolic process	7	0.00364
	GO.0072594	establishment of protein localization to organelle	6	0.0119
	GO.0006605	protein targeting	6	0.0223
	GO.0006886	intracellular protein transport	7	0.0329
	GO.0006749	glutathione metabolic process	3	0.0425
	GO.0033365	protein localization to organelle	6	0.0425
	GO.1902582	single-organism intracellular transport	8	0.0425
	GO.0006520	cellular amino acid metabolic process	5	0.0434
	GO.0006790	sulfur compound metabolic process	5	0.0434
	GO.1902580	single-organism cellular localization	7	0.0434
	GO.0006979	response to oxidative stress	5	0.0468
Cellular Component (GO)	GO.0005739	mitochondrion	10	0.00523
	GO.0005777	peroxisome	4	0.00523
	GO.0005829	cytosol	13	0.00523
	GO.0022625	cytosolic large ribosomal subunit	3	0.00826
	GO.0005840	ribosome	4	0.0215
	GO.0022626	cytosolic ribosome	3	0.0338
KEGG pathway	4146	Peroxisome	3	0.0331

Table S8. STRING analysis result of up- regulated and oxidized outlying proteins in A549 cells.

	pathway ID	pathway description	observed gene count	false discovery rate
Biological Process (GO)	GO:0042254	ribosome biogenesis	5	0.00037
	GO:0034641	cellular nitrogen compound metabolic process	11	0.0083
	GO:0006412	translation	4	0.01
	GO:0006413	translational initiation	3	0.0102
	GO:0044271	cellular nitrogen compound biosynthetic process	9	0.0102
	GO:0006364	rRNA processing	3	0.0157
	GO:0006518	peptide metabolic process	4	0.0157
	GO:0010501	RNA secondary structure unwinding	2	0.0163
	GO:0045815	positive regulation of gene expression, epigenetic	2	0.019
	GO:0002181	cytoplasmic translation	2	0.0218
	GO:0006614	SRP-dependent cotranslational protein targeting to membrane	2	0.0286
	GO:0006725	cellular aromatic compound metabolic process	9	0.0286
	GO:0006807	nitrogen compound metabolic process	12	0.0286
	GO:0010467	gene expression	8	0.0286
	GO:0034645	cellular macromolecule biosynthetic process	8	0.0286
	GO:0042273	ribosomal large subunit biogenesis	2	0.0286
	GO:0044237	cellular metabolic process	12	0.0286
	GO:0044238	primary metabolic process	12	0.0286
	GO:0046483	heterocycle metabolic process	9	0.0286
	GO:1901566	organonitrogen compound biosynthetic process	5	0.0286
	GO:1901576	organic substance biosynthetic process	9	0.0286
	GO:1901360	organic cyclic compound metabolic process	9	0.0288
	GO:0072594	establishment of protein localization to organelle	3	0.0292
	GO:0072524	pyridine-containing compound metabolic process	2	0.0318
	GO:0000184	nuclear-transcribed mRNA catabolic process, nonsense-mediated decay	2	0.0331
	GO:0071704	organic substance metabolic process	12	0.0331
	GO:0071840	cellular component organization or biogenesis	9	0.0393
Molecular Function (GO)	GO:0003735	structural constituent of ribosome	3	0.0167
	GO:0004004	ATP-dependent RNA helicase activity	2	0.0495
	GO:0005198	structural molecule activity	4	0.0495
	GO:0019843	rRNA binding	2	0.0495
	GO:1901363	heterocyclic compound binding	9	0.0499
	GO:0003676	nucleic acid binding	7	0.05
	GO:0097159	organic cyclic compound binding	9	0.05
Cellular Component (GO)	GO:0005840	ribosome	4	0.0011
	GO:0015934	large ribosomal subunit	3	0.0016
	GO:0022625	cytosolic large ribosomal subunit	2	0.0064
	GO:0043232	intracellular non-membrane-bounded organelle	8	0.0394
KEGG pathways	hsa03010	Ribosome	3	0.0027

Table S9. STRING analysis result of up- regulated and reduced outlying proteins in A549 cells.

	pathway ID	pathway description	observed gene count	false discovery rate
**Biological Process (GO)**	GO:0034249	negative regulation of cellular amide metabolic process	3	0.046
**Molecular Function (GO)**	GO:0005783	endoplasmic reticulum	7	0.0022
	GO:0044444	cytoplasmic part	11	0.0258
	GO:0005844	polysome	2	0.0286
	GO:0012505	endomembrane system	8	0.0286
	GO:1990904	ribonucleoprotein complex	4	0.0286
**KEGG Pathways**	hsa04216	Ferroptosis	2	0.0083

Table S10. STRING analysis result of down- regulated and reduced outlying proteins in A549 cells.

	pathway ID	pathway description	observed gene count	false discovery rate
**Biological Process (GO)**	GO:0022607	cellular component assembly	11	0.0047
	GO:1903078	positive regulation of protein localization to plasma membrane	3	0.0088
	GO:0007015	actin filament organization	4	0.0094
	GO:0016043	cellular component organization	14	0.0094
	GO:1904951	positive regulation of establishment of protein localization	5	0.0094
	GO:0065003	protein-containing complex assembly	8	0.0095
	GO:0030029	actin filament-based process	5	0.0116
	GO:0030048	actin filament-based movement	3	0.0144
	GO:1903829	positive regulation of cellular protein localization	4	0.0148
	GO:0006928	movement of cell or subcellular component	7	0.017
	GO:0034249	negative regulation of cellular amide metabolic process	3	0.0205
	GO:0034329	cell junction assembly	3	0.0205
	GO:0051222	positive regulation of protein transport	4	0.0247
	GO:0071702	organic substance transport	8	0.0274
	GO:0030049	muscle filament sliding	2	0.0326
	GO:0033036	macromolecule localization	8	0.0424
	GO:0051017	actin filament bundle assembly	2	0.0424
	GO:0065008	regulation of biological quality	10	0.0424
	GO:0032663	regulation of interleukin-2 production	2	0.0452
**Molecular Function (GO)**	GO:0003779	actin binding	5	0.0096
	GO:0008092	cytoskeletal protein binding	6	0.0178
	GO:0051015	actin filament binding	3	0.0348
	GO:0008307	structural constituent of muscle	2	0.0484
**Cellular Component (GO)**	GO:0044444	cytoplasmic part	20	9.47E-05
	GO:0005829	cytosol	15	0.00041
	GO:0015629	actin cytoskeleton	6	0.00041
	GO:0005783	endoplasmic reticulum	9	0.0014
	GO:0030016	myofibril	4	0.0023
	GO:0043229	intracellular organelle	20	0.0023
	GO:0044449	contractile fiber part	4	0.0023
	GO:0099512	supramolecular fiber	6	0.0038
	GO:0044446	intracellular organelle part	17	0.0052
	GO:0043209	myelin sheath	3	0.0079
	GO:0030017	sarcomere	3	0.0138
	GO:0001725	stress fiber	2	0.0157
	GO:0005844	polysome	2	0.0207
	GO:0005884	actin filament	2	0.0207
	GO:0097458	neuron part	6	0.0229
	GO:0030863	cortical cytoskeleton	2	0.0247
	GO:0005856	cytoskeleton	7	0.0256
	GO:0032991	protein-containing complex	11	0.0256
	GO:0043232	intracellular non-membrane-bounded organelle	10	0.0256
	GO:0044430	cytoskeletal part	6	0.0259
	GO:0031253	cell projection membrane	3	0.0278
	GO:0005903	brush border	2	0.0292
	GO:0030175	filopodium	2	0.0292
	GO:0048471	perinuclear region of cytoplasm	4	0.0292
	GO:0043005	neuron projection	5	0.0313
	GO:0012505	endomembrane system	10	0.0353
	GO:0031252	cell leading edge	3	0.0363
	GO:1990904	ribonucleoprotein complex	4	0.0395
	GO:0005911	cell-cell junction	3	0.042
	GO:0098794	postsynapse	3	0.0487
	GO:0120025	plasma membrane bounded cell projection	6	0.0487
	GO:0120038	plasma membrane bounded cell projection part	5	0.0487
	GO:0031256	leading edge membrane	2	0.0495
**KEGG Pathways**	hsa04216	Ferroptosis	2	0.0269
	hsa04530	Tight junction	3	0.0269

Table S11. STRING analysis result of down- regulated and oxidized outlying proteins in A549 cells.

	pathway ID	pathway description	observed gene count	false discovery rate
Biological Process (GO)	GO:0045454	cell redox homeostasis	6	1.21E-08
	GO:0019725	cellular homeostasis	7	0.00029
	GO:0098869	cellular oxidant detoxification	4	0.00029
	GO:0042743	hydrogen peroxide metabolic process	3	0.00038
	GO:0032781	positive regulation of ATPase activity	3	0.0015
	GO:0051186	cofactor metabolic process	5	0.0023
	GO:0051881	regulation of mitochondrial membrane potential	3	0.0023
	GO:0007568	aging	4	0.0032
	GO:0055114	oxidation-reduction process	6	0.0032
	GO:0019430	removal of superoxide radicals	2	0.0042
	GO:0017144	drug metabolic process	5	0.0046
	GO:0034614	cellular response to reactive oxygen species	3	0.006
	GO:0042744	hydrogen peroxide catabolic process	2	0.006
	GO:0006979	response to oxidative stress	4	0.0066
	GO:0042391	regulation of membrane potential	4	0.0089
	GO:1900027	regulation of ruffle assembly	2	0.0089
	GO:0019752	carboxylic acid metabolic process	5	0.0125
	GO:0065008	regulation of biological quality	9	0.0125
	GO:0090066	regulation of anatomical structure size	4	0.0125
	GO:0010035	response to inorganic substance	4	0.0136
	GO:0009069	serine family amino acid metabolic process	2	0.0139
	GO:0006457	protein folding	3	0.0153
	GO:0030837	negative regulation of actin filament polymerization	2	0.0153
	GO:0051188	cofactor biosynthetic process	3	0.0153
	GO:0060548	negative regulation of cell death	5	0.0153
	GO:0006749	glutathione metabolic process	2	0.0183
	GO:0046686	response to cadmium ion	2	0.0202
	GO:0032233	positive regulation of actin filament bundle assembly	2	0.0221
	GO:0080135	regulation of cellular response to stress	4	0.0223
	GO:0044093	positive regulation of molecular function	6	0.0233
	GO:0061077	chaperone-mediated protein folding	2	0.0233
	GO:1903201	regulation of oxidative stress-induced cell death	2	0.0238
	GO:0007422	peripheral nervous system development	2	0.0255
	GO:0044281	small molecule metabolic process	6	0.0269
	GO:0006520	cellular amino acid metabolic process	3	0.0294
	GO:0051492	regulation of stress fiber assembly	2	0.0308
	GO:0030838	positive regulation of actin filament polymerization	2	0.0325
	GO:0051336	regulation of hydrolase activity	5	0.0325
	GO:0043603	cellular amide metabolic process	4	0.0334
	GO:0006790	sulfur compound metabolic process	3	0.0352
	GO:0009408	response to heat	2	0.0352
	GO:0048523	negative regulation of cellular process	9	0.0352
	GO:0043648	dicarboxylic acid metabolic process	2	0.0399
	GO:0002262	myeloid cell homeostasis	2	0.0433
	GO:0043085	positive regulation of catalytic activity	5	0.0443
	GO:0033138	positive regulation of peptidyl-serine phosphorylation	2	0.0445
	GO:0042542	response to hydrogen peroxide	2	0.0497
	GO:0043066	negative regulation of apoptotic process	4	0.0497
Molecular Function (GO)	GO:0016209	antioxidant activity	4	6.40E-05
	GO:0016668	oxidoreductase activity, acting on a sulfur group of donors, NAD(P) as acceptor	3	6.40E-05
	GO:0008379	thioredoxin peroxidase activity	2	0.00057
	GO:0016491	oxidoreductase activity	6	0.00057
	GO:0003824	catalytic activity	12	0.0014
	GO:0003785	actin monomer binding	2	0.0045
	GO:0060590	ATPase regulator activity	2	0.0077
	GO:0005546	phosphatidylinositol-4,5-bisphosphate binding	2	0.0161
	GO:0051087	chaperone binding	2	0.0329
	GO:0051082	unfolded protein binding	2	0.0345
Cellular Component (GO)	GO:0043209	myelin sheath	5	1.50E-05
	GO:0042470	melanosome	3	0.0052
	GO:0032839	dendrite cytoplasm	2	0.0075

	GO:0031410	cytoplasmic vesicle	7	0.0205
	GO:0044444	cytoplasmic part	13	0.0374
	GO:0005737	cytoplasm	14	0.0436
	GO:0099568	cytoplasmic region	3	0.0444
**KEGG Pathways**	hsa00010	Glycolysis / Gluconeogenesis	2	0.0097
	hsa00280	Valine, leucine and isoleucine degradation	2	0.0097
	hsa00620	Pyruvate metabolism	2	0.0097
	hsa04141	Protein processing in endoplasmic reticulum	3	0.0097
	hsa04216	Ferroptosis	2	0.0097
	hsa05131	Shigellosis	2	0.0097
	hsa04146	Peroxisome	2	0.0103
	hsa05132	Salmonella infection	2	0.0103
	hsa04015	Rap1 signaling pathway	2	0.0465
	hsa04810	Regulation of actin cytoskeleton	2	0.0465

Table S17. Oxidation state of most significantly oxidized and reduced proteins in A549 cells grown in DEW, NW and DDW.

Protein names	Gene names	Oxidation % (median)			DEW/NW	DDW/NW
		DEW	NW	DDW		
Tyrosine-protein kinase BAZ1B	BAZ1B	17.80	5.83	1.61	3.05	0.28
60S acidic ribosomal protein P0	RPLP0	57.13	9.21	8.47	6.20	0.92
DNA polymerase alpha subunit B	POLA2	30.06	6.98	8.05	4.31	1.15
Fatty aldehyde dehydrogenase	ALDH3A2	29.28	5.79	6.59	5.06	1.14
Xaa-Pro dipeptidase	PEPD	35.88	10.03	9.80	3.58	0.98
Dihydrolipoyl dehydrogenase, mitochondrial	DLD	30.81	9.48	9.02	3.25	0.95
Protein arginine N-methyltransferase 3	PRMT3	56.60	11.10	16.71	5.10	1.51
Probable 28S rRNA (cytosine(4447)-C(5))-methyltransferase	NOP2	39.09	21.87	10.60	1.79	0.48
Guanine nucleotide-binding protein G(I)/G(S)/G(T) subunit beta-2	GNB2	-	-	-	-	-
Thioredoxin	TXN	20.06	5.93	6.00	3.38	1.01
Ribosomal RNA processing protein 1 homolog B	RRP1B	34.10	22.05	10.51	1.55	0.48
60S ribosomal protein L10a	RPL10A	-	-	-	-	-
Isocitrate dehydrogenase [NADP] cytoplasmic	IDH1	20.91	8.86	6.66	2.36	0.75
Serine--tRNA ligase, cytoplasmic	SARS	20.99	5.75	5.91	3.65	1.03
Probable ATP-dependent RNA helicase DDX27	DDX27	26.66	9.49	8.69	2.81	0.92
Peroxiredoxin-5, mitochondrial	PRDX5	68.88	26.74	19.82	2.58	0.74
Glutamate--cysteine ligase catalytic subunit	GCLC	11.07	3.49	3.77	3.17	1.08
Importin-7	IPO7	16.12	7.20	6.57	2.24	0.91
60S ribosomal protein L9	RPL9	30.30	5.59	12.00	5.42	2.15
Coatomer subunit delta	ARCN1	-	-	-	-	-
Superoxide dismutase [Cu-Zn]	SOD1	-	-	-	-	-
Nucleolar RNA helicase 2	DDX21	10.95	7.36	4.14	1.49	0.56
WD repeat-containing protein 43	WDR43	19.54	6.59	7.61	2.96	1.15
Histidine--tRNA ligase, cytoplasmic	HARS	18.37	8.52	7.25	2.16	0.85
ADP/ATP translocase 3	SLC25A6	15.28	6.73	6.55	2.27	0.97
Fascin	FSCN1	2.25	10.50	45.35	0.21	4.32
Plasma membrane calcium-transporting ATPase 1	ATP2B1	-	-	-	-	-
SEC23-interacting protein	SEC23IP	-	-	-	-	-
T-complex protein 1 subunit eta	CCT7	3.16	13.33	20.23	0.24	1.52
Spectrin beta chain, non-erythrocytic 1	SPTBN1	2.68	8.11	13.12	0.33	1.62
Leucine--tRNA ligase, cytoplasmic	LARS	2.74	9.08	13.21	0.30	1.46
Alpha-actinin-4	ACTN4	1.92	4.84	8.60	0.40	1.78
Vasodilator-stimulated phosphoprotein	VASP	6.37	13.59	23.73	0.47	1.75
Splicing factor 3B subunit 1	SF3B1	5.82	16.14	23.65	0.36	1.47
40S ribosomal protein S3a	RPS3A	2.02	9.68	8.49	0.21	0.88
Elongation factor 1-alpha 1	EEF1A1	1.28	6.48	7.65	0.20	1.18
T-complex protein 1 subunit gamma	CCT3	2.35	5.64	6.45	0.42	1.14
RNA-binding protein 14	RBM14	3.98	10.14	16.43	0.39	1.62
Plastin-3	PLS3	2.58	9.61	9.13	0.27	0.95
Myosin light polypeptide 6	MYL6	5.11	13.20	17.87	0.39	1.35
Methionine--tRNA ligase, cytoplasmic	MARS	3.24	8.16	11.22	0.40	1.38
26S proteasome non-ATPase regulatory subunit 9	PSMD9	-	-	-	-	-
Phosphatidylinositol transfer protein beta isoform	PITPNB	8.51	12.98	27.86	0.66	2.15
Dynein light chain 1, cytoplasmic	DYNLL1	3.26	5.78	14.11	0.56	2.44
Isocitrate dehydrogenase [NAD] subunit alpha, mitochondrial	IDH3A	6.17	4.46	16.73	1.38	3.75
60S ribosomal protein L12	RPL12	5.41	11.89	19.31	0.46	1.62
Serine/threonine-protein phosphatase PP1-alpha catalytic subunit;Serine/threonine-protein phosphatase	PPP1CA	2.74	4.54	8.07	0.60	1.78
Plasminogen activator inhibitor 1 RNA-binding protein	SERBP1	4.53	9.71	13.16	0.47	1.36
Actin-related protein 2	ACTR2	6.70	6.14	24.07	1.09	3.92
RNA polymerase II-associated protein 3	RPAP3	13.28	12.10	42.27	1.10	3.49

"-": proteins were not identified in all datasets

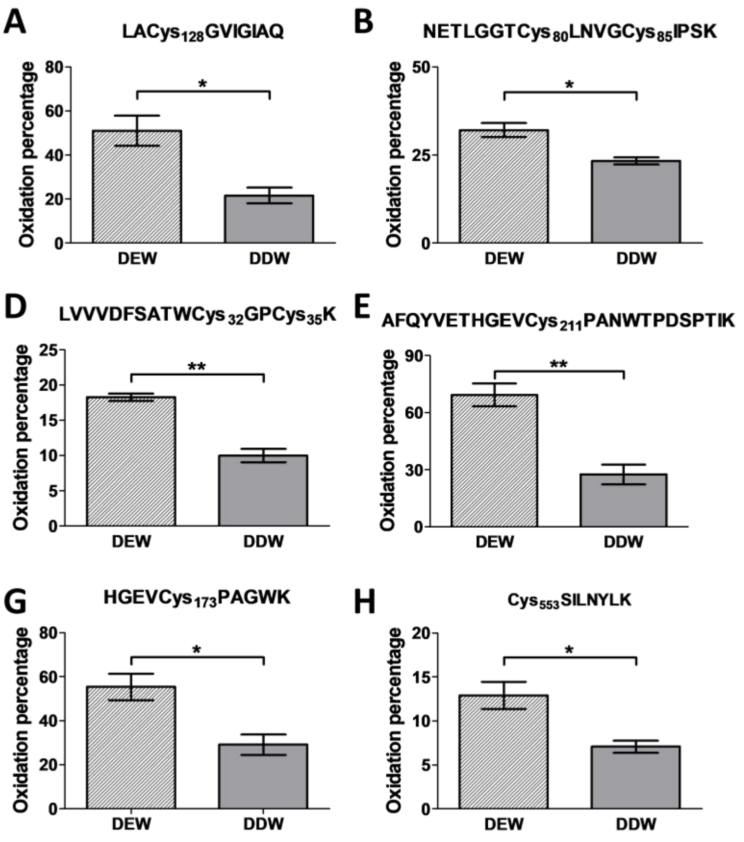

Figure S1. Oxidized percentage of cysteines in the peptides in A549 cells for DEW vs DDW. A-H show mean ± standard error in three replictes, * - $p<0.05$,** - $p<0.01$ in two-tailed unpaired t-test.

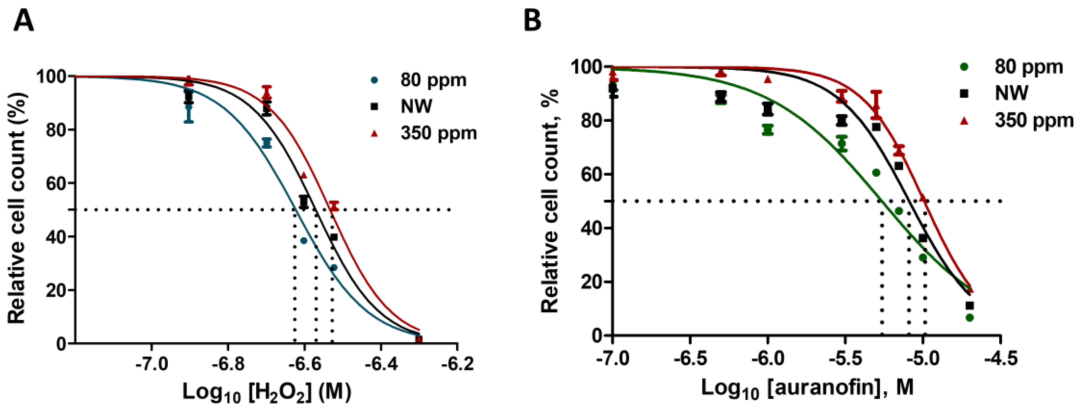

Figure S2. Survival curves of A549 cells in DDW, NW or DEW treated with A) H_2O_2, B) auranofin. A-B show mean ± standard error in four replicates. The survival curves were fitted by log(inhibitor) vs. normalized response - Variable slope in GraphPad Prism.

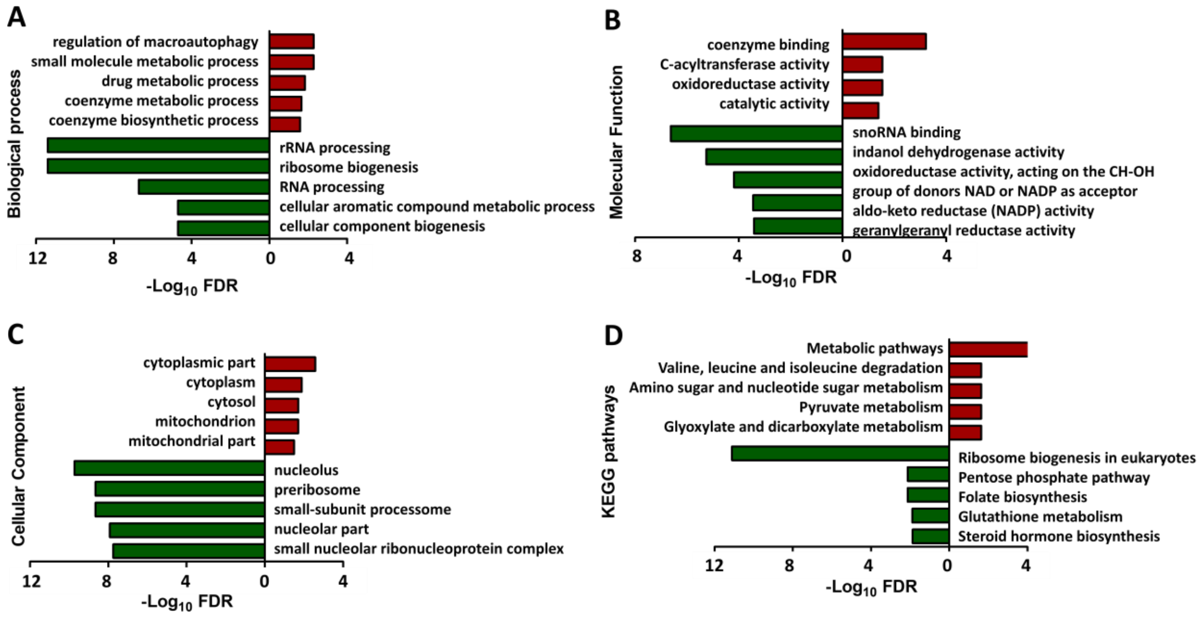

Figure S3. STRING network analysis of significantly regulated proteins in HT29 cells grown in 450 ppm DEW vs 100 ppm DDW. GO biological process (A), molecular function (B), cellular component (C) and KEGG pathways (D) analysis of top 30 up- (red) and down- (green) regulated proteins.

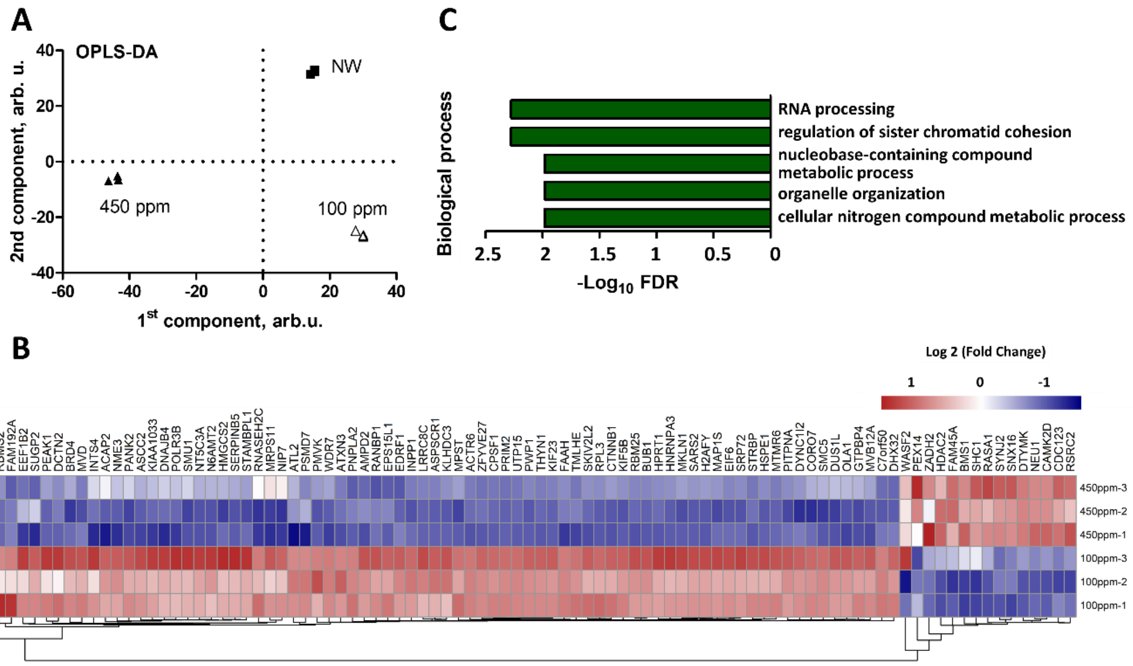

Figure S4. Redox proteomics analysis of deuterium effect on HT29 cells. A) OPLS-DA of redox datasets. B) Heat map of significantly reduced (fold change < 0.5, n = 78) and oxidized (fold change > 2, n = 15) proteins. C) Biological process analysis of significantly reduced proteins in HT29 cells grown in 450 ppm DEW compared to 100 ppm DDW.
